# Causal relationship between genetically predicted depression and cancer risk: a two-sample bi-directional mendelian randomization

**DOI:** 10.1186/s12885-022-09457-9

**Published:** 2022-03-31

**Authors:** Guang-Li Zhu, Cheng Xu, Kai-bin Yang, Si-Qi Tang, Ling-Long Tang, Lei Chen, Wen-Fei Li, Yan-Ping Mao, Jun Ma

**Affiliations:** Department of Radiation Oncology, Sun Yat-Sen University Cancer Center State Key Laboratory of Oncology in South China, Collaborative Innovation Center for Cancer Medicine, Guangdong Key Laboratory of Nasopharyngeal Carcinoma Diagnosis and Therapy, 510060 Guangzhou, PR China

**Keywords:** Depression, Cancer, Mendelian randomization, Causality, GWAS

## Abstract

**Background:**

Depression has been reported to be associated with some types of cancer in observational studies. However, the direction and magnitude of the causal relationships between depression and different types of cancer remain unclear.

**Methods:**

We performed the two-sample bi-directional mendelian randomization with the publicly available GWAS summary statistics to investigate the causal relationship between the genetically predicted depression and the risk of multiple types of cancers, including ovarian cancer, breast cancer, lung cancer, glioma, pancreatic cancer, lymphoma, colorectal cancer, thyroid cancer, bladder cancer, and kidney cancer. The total sample size varies from 504,034 to 729,150. Causal estimate was calculated by inverse variance weighted method. We also performed additional sensitivity tests to evaluate the validity of the causal relationship.

**Results:**

After correction for heterogeneity and horizontal pleiotropy, we only detected suggestive evidence for the causality of genetically predicted depression on breast cancer (OR = 1.09, 95% CI: 1.03–1.15, *P* = 0.0022). The causal effect of depression on breast cancer was consistent in direction and magnitude in the sensitivity analysis. No evidence of causal effects of depression on other types of cancer and reverse causality was detected.

**Conclusions:**

The result of this study suggests a causative effect of genetically predicted depression on specific type of cancer. Our findings emphasize the importance of depression in the prevention and treatment of breast cancer.

**Supplementary Information:**

The online version contains supplementary material available at 10.1186/s12885-022-09457-9.

## Background

Depression is the most common mental illness worldwide. The incidence of depression worldwide increased by 49.86% from 1990 to 2017 [1]. As estimated by World Health Organization (WHO), depression has been affecting over 300 million people by 2015, which accounts for 4.4% of the global population [2]. WHO predicted that depression would rank first among all the causes of burden of disease worldwide by 2030. As depression impairs both mental and physical health, it has become an important public health problem and a leading cause of disability worldwide nowadays [3].

Depression has been reported to be associated with many physical diseases, such as cardiovascular disease [4]. For a long time, depression had been recognized as a comorbidity of cancer, rather than a risk factor of cancer. In recent years, the causal relationships between depression and cancer risk have been widely explored in many observational studies. However, their results were controversial. Some observational studies suggested that causal relationship exists between depression and cancer risk [5–8], while others did not [9–11]. Meanwhile, a recent meta-analysis reported a small and positive association between depression and risk of overall cancer [12], as well as risk of lung cancer and liver cancer, while a previous meta-analysis did not [13]. The reasons of these controversies might be that the settings in these studies vary greatly, including the types of cancer and the controlled confounding factors. Since the inference of causal relationships in observational studies is usually confronted with the challenge of potential confounding bias and reverse causality, the association between depression and cancer remained to be elucidated. Although the best approach for causal inference is the randomized controlled trial, it is not feasible in the causal inference for depression as exposure, because depression cannot be randomized to different groups of individuals.

Mendelian randomization (MR) analysis is a promising tool for causal inference under the background of rapid development of large-scale GWAS [14]. It utilizes the genetic variants strongly associated with exposure as instrumental variables to explore the causal relationship between exposures and outcomes. The MR analysis depends on the natural randomized assortment of genetic variants. According to the principle of mendelian inheritance, each parent randomly contributes one allele for each gene to its offspring. This process is independent of confounders. Thus, the MR analysis provides an analogue for randomized controlled trials. A genetic variable is valid in the MR analysis if it meets the following 3 assumptions: i) the genetic variants are associated with exposure; ii) the genetic variants are independent of confounders between exposure and outcomes; iii) the genetic variants only influence outcome via exposure [15]. The last assumption is also known as the no-pleiotropy assumptions or exclusion-restriction principle, which means that the genetic variants cannot act on outcome via other alternative causal pathways that exclude exposure. A two-sample MR analysis refers to the MR analysis which included a pair of exposure and outcome from different or non-overlapping populations, and a bi-directional MR analysis tries to explore the reverse causality.

In this study, we performed the two-sample bi-directional MR with publicly available GWAS summary statistics to explore the causal relationship between depression and the risk of multiple types of cancers, including ovarian cancer, breast cancer, lung cancer, glioma, pancreatic cancer, lymphoma, colorectal cancer, thyroid cancer, bladder cancer, and kidney cancer. The selection of the types of cancer for analysis depends on the public availability of their GWAS data. The illustration of the causal relationships between depression and cancer will contribute to the prevention and treatment of these diseases.

## Methods

### Data source of depression

Summary statistics for depression were retrieved from the largest GWAS meta-analysis for depression up to date, which were conducted by Howard et al. [16]. It consists of three large-scale GWAS including 23andMe, Psychiatric Genomics Consortium (PGC) and UK Biobank, which included 807,553 individuals in total (246,363 cases and 561,190 controls). Hyde et al. used self-reported data of clinical diagnosis of depression through web-based surveys from 23andMe, Inc., a consumer genetics company, providing a total of 75,607 cases and 231,747 controls (*n* = 307,354) for analysis [17]. Within UK Biobank, Howard et al. used the broad definition of depression defined by the participants’ response to the questions ‘Have you ever seen a general practitioner for nerves, anxiety, tension or depression?’ or ‘Have you ever seen a psychiatrist for nerves, anxiety, tension or depression?’, providing a total of 127,552 cases and 233,763 controls (*n* = 361,315) for analysis. Within PGC cohorts, depression should be diagnosed by international consensus criteria (DSM-IV, ICD-9, or ICD-10), and the cohorts provided a total of 12,149,399 variant calls for 43,204 cases and 95,680 controls (*n* = 138,884) for analysis. The participants from the cohorts above were all European ancestry. 102 independent SNPs associated with depression were identified in this meta-analysis. Among these three GWAS, the summary statistics for all assessed genetic variants were only publicly available for UK Biobank and PGC, so we included the full summary statistics from 2 cohorts, PGC and UK Biobank, provided by Howard et al. to perform bi-directional MR analysis. Considering that the exclusion of data of the 23andMe cohort from MR analysis might lower the power, we utilized the summary statistics of depression as exposure from the meta-analysis of 23andMe, PGC and UK Biobank cohorts as a replication set for sensitivity analysis to explore the validity of the causal effect of depression on certain types of cancer.

### Data source of different types of cancer

The summary statistics from GWAS for multiple kinds of cancers in publicly available databases were retrieved from MRC IEU OpenGWAS (MR-base) database [18]. The two-sample MR method requires two independent samples from the same population. If the population of the GWAS of cancers were not European ancestry, such GWAS will be excluded. Besides, to reduce the bias caused by overlapping datasets of exposure and outcome, if the GWAS for cancer included participants of the UK biobank, such GWAS will also be excluded.

Supplementary Table S1 presents the summary of the data source of different traits, including number of SNPs, number of cases, number of controls, sample size, etc. The estimates for the association between the genetic variants and risk of ovarian, breast, lung, glioma, and pancreatic cancer were obtained, respectively, from the publicly available summary statistics of Ovarian Cancer Association Consortium (OCAC) [19], Breast Cancer Association Consortium (BCAC) [20], International Lung Cancer Consortium (ILCCO) [21], Cohort-Based Genome-Wide Association Study of Glioma (GliomaScan) [22], and Pancreatic Cancer Cohort Consortium (PanScan) [23]. The estimates for the association between the genetic variants and risk of lymphoma, colorectal cancer, thyroid cancer, bladder cancer, and kidney cancer excluding renal pelvis were obtained, respectively, from the publicly available summary statistics of FinnGen consortium (www.finbb.fi). The above studies included participants of European ancestry only.

As the data included in this study is publicly available, we did not apply for any specific ethical consent or review from any participants of the GWAS above.

### Statistical analysis

**Fig. 1:**
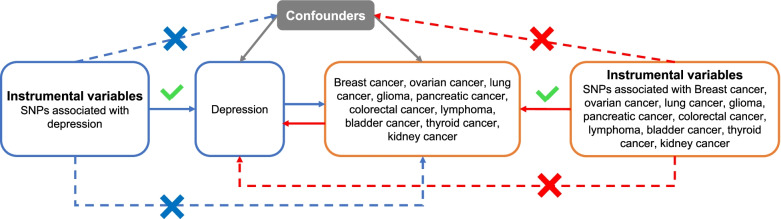
Study design of the bidirectional mendelian randomization between depression and different types of cancer. The blue solid lines represent the association between the instrumental variables (SNPs) and exposure as well as the association between exposure and outcome. The red solid lines represent the association of reverse causality. Dash lines with cross means that the association meets two basic assumption of mendelian randomization: i) the genetic variants (SNPs) are independent of confounders between exposure and outcomes; ii) the genetic variants only influence outcome via exposure

To assess the causal relationship between depression and multiple kinds of cancers, we conducted a bidirectional two-sample MR analysis for each pair of exposure and outcome. Figure 1 presents the workflow of our study.

For depression as exposure, we utilized 96 out of the 102 independent SNPs identified in the meta-analysis by Howard et al. as genetic instruments [16]. Meanwhile, for a certain type of cancer as exposure, we selected the genome-wide statistically significant (*P* < 5 × 10^−8^) SNPs associated with this type of cancer from the corresponding GWAS. To mitigate the bias caused by linkage disequilibrium (LD), we clumped the SNPs within 5 kb and sharing a LD with *r*^2^ > 0.001 together, and only selected the SNPs with the strongest effect on exposure as genetic instruments.

The summary statistics of these SNPs were retrieved from the GWAS meta-analysis for depression by Howard et al. and the GWAS of different types of cancer respectively. We tried to find a proxy SNP with high LD (*r*^2^ > 0.8) for those SNPs without matched records in the GWAS or meta-analysis of GWAS of outcome. Finally, these SNPs were excluded from analysis if no proxy SNP could be identified. Supplementary Tables S2 and S8 present all SNPs included in the MR analysis of each pair of exposure and outcome.

We used the conventional fixed-effect inverse-variance weighted (IVW) method to estimate the causal effect of exposure on outcomes [24]. For those MR analyses with high variant heterogeneity measured by the Cochran’s Q statistics, we used the random-effect IVW method to correct for the heterogeneity [25]. For those exposures with only one associated SNP as genetic instrument, we use Wald ratio method to estimate the causal effect. IVW is the most efficient MR method with the greatest statistical power, but it assumes that all instrumental variables are valid, and it will be biased if the average pleiotropic effects differ from zero. Weighted median method is more robust to outliers and only assumes that the majority of the instrumental variables are valid [26]. Thus, we performed sensitivity analysis to assess the robustness of the estimate of causal effect, including the weighted median method [27], the leave-one-out sensitivity test [28], and the Steiger filtering [29]. In Steiger filtering, we first calculated *R*^2^, the proportion of variance in the exposures and outcomes explained by SNPs, and the SNPs that explained less variance in exposures than that in outcomes were filtered. Causal effect estimation with IVW method was repeated after filtering. We also performed MR directionality Steiger test to confirm whether the direction of effect is oriented from exposure to outcome.For exposures with at least 5 associated SNPs as genetic instruments, we used MR Egger intercept test [30] to evaluate the horizontal pleiotropy across all genetic instruments. However, it is sensitive to outliers and violations of INstrument Strength Independent of Direct Effect (INSIDE) assumption, thus less efficient. Therefore, we also conducted MR pleiotropy residual sum and outlier (MR-PRESSO) global test [31], which is more robust to outliers [26]. Furthermore, where there was any evidence of horizontal pleiotropy, we performed MR-PRESSO outlier test which detects genetic instruments of horizontal pleiotropy as outliers and provides the estimate of causal effect again after the removal of outliers based on IVW method. We also performed MR-PRESSO distortion test to detect whether there was statistically significant difference in the estimate of causal effect before and after removal of outliers.

The conclusion of causality will be drawn if it shows consistent direction and estimate of causal effect in IVW and weighted median method, right orientation of causal relationship confirmed by Steiger test, and a *P*-value of IVW method less than the Bonferroni-corrected significance level of 1.2 × 10^−3^ (*P*-value threshold = 0.05/43: corrected for 43 pairs of exposure and outcome) after the correction for heterogeneity and horizontal pleiotropy. A *P*-value between 1.2 × 10^−3^ and 0.05 will be considered as suggestive evidence of causality.

### Power and F-statistics calculation

We first calculated the power for our IVW analyses using an online web tool (http://cnsgenomics.com/shiny/mRnd/) [32], in which type-I error rate (α = 0.05), corresponding proportion of cases in the study (Supplementary Table S1) and point estimate of odds ratio calculated by fixed-effect IVW method (Supplementary Tables S3 and S9) were also used. F-statistics equals to ((N − k − 1)/k) * (*R*^2^ /(1 − *R*^2^)), in which N and k denotes the sample size and number of SNPs respectively [33]. F-statistics is the measurement of the strength of genetic instruments. A F-statistics less than 10 usually indicates the weak instrument bias.

All statistical analyses were performed with the MR-Base ‘TwoSampleMR’ v0.5.5 package, “MRPRESSO” v1.0 package (R Foundation for Statistical Computing, Vienna, Austria).

## Results

### Causal effect of depression on cancer

**Fig. 2:**
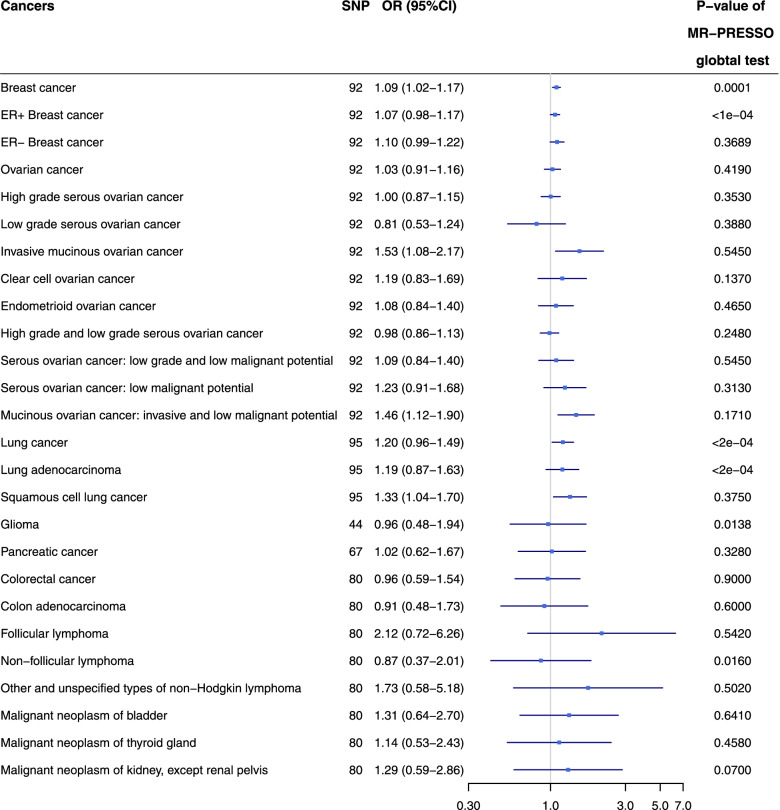
The causal estimates of depression on different types of cancer and the evaluation of their horizontal pleiotropy by MR-PRESSO. MR-PRESSO: Mendelian randomization-pleiotropy residual sum and outlier

Figure 2 and Supplementary Table S3 present the results of MR analysis of causal effect of depression on different types of cancer and the evaluation of pleiotropy effect. We also provided scatter and funnel plot of each pair of association for better demonstration of causality and identification of heterogeneity (Supplementary Figures S1 and S2). In the primary MR analysis, the genetic instruments included in each pair of exposure and outcome varied from 44 to 95. The maximal proportion of variance in depression explained by SNPs was 0.415%. The maximal F-statistics of depression was 21.7. Suggestive evidence of causality was detected in depression on breast cancer (OR = 1.09, 95% CI: 1.03–1.15, *P* = 0.0022), invasive mucinous ovarian cancer (OR = 1.53, 95% CI: 1.08–2.17, *P* = 0.0177), invasive and low malignant potential mucinous ovarian cancer (OR = 1.46, 95% CI: 1.12–1.90, *P* = 0.0057), lung cancer (OR = 1.20, 95% CI: 1.02–1.40, *P* = 0.0244) and squamous cell lung cancer (OR = 1.33, 95% CI: 1.04–1.70, *P* = 0.0207) in MR analysis with the fixed-effect IVW method. Among these five types of cancer, heterogeneity was detected in breast cancer (*P* = 1.0 × 10^–4^) and lung cancer (*P* = 1.50 × 10^–7^). After correcting for heterogeneity with random-effect method, the causal effect of depression on lung cancer (OR = 1.20, 95% CI: 0.96–1.49, *P* = 0.1055) was no longer statistically significant, while breast cancer remained similar (OR = 1.09, 95% CI: 1.02–1.17, *P* = 0.0176). After excluding lung cancer, among the remaining four types of cancer with suggestive evidence of causality, we detected horizontal pleiotropy in breast cancer (*P* = 1.0 × 10^–4^) by MR-PRESSO global test. After the removal of two outlier SNPs, the estimate of causal effect of depression on breast cancer (OR = 1.10, 95% CI: 1.03–1.16, *P* = 0.0072) remains similar, and the MR-PRESSO distortion test is not statistically significant (*P* = 0.9518) (Supplementary Table S4).

In the sensitivity analysis, we demonstrated similar findings in breast cancer (OR = 1.09, 95% CI: 1.03–1.15, *P* = 0.0037), invasive mucinous ovarian cancer (OR = 1.54, 95% CI: 1.08–2.20, *P* = 0.0170), invasive and low malignant potential mucinous ovarian cancer (OR = 1.44, 95% CI: 1.10–1.88, *P* = 0.0081), and squamous cell lung cancer (OR = 1.35, 95% CI: 1.06–1.72, *P* = 0.0168) in the 3-cohort replication set including PGC, UKB and 23andMe (Supplementary Table S3). Besides, in the sensitivity analysis with weighted median method, the causal effect of depression on invasive and low malignant potential mucinous ovarian cancer (OR = 1.51, 95% CI: 1.01–2.26), squamous cell lung cancer (OR = 1.43, 95% CI: 1.01–2.03) and breast cancer (OR = 1.09, 95% CI: 1.00–1.19) agreed in direction and magnitude with IVW method. The leave-one-out analysis revealed that the causal estimates were not driven by a particular SNP (Supplementary Table S5, Supplementary Figures S5-S30). However, after Steiger filtering, the causal relationship between depression and invasive mucinous ovarian cancer (OR = 1.04, 95% CI: 0.57–1.90, *P* = 0.8900), invasive and low malignant potential mucinous ovarian cancer (OR = 1.05, 95% CI: 0.72–1.54, *P* = 0.7824), and squamous cell lung cancer (OR = 0.99, 95% CI: 0.72–1.37, *P* = 0.9689) no longer existed (Supplementary Table S6). Only breast cancer showed correct Steiger direction (*P* < 0.0001). Given the sample size of these types of cancer, the statistical power for breast cancer was 65% (Supplementary Table S7), while the statistical power for the other types of cancer varied from 5 to 100% (Supplementary Table S7).

### Causal effect of cancer on depression

**Fig. 3:**
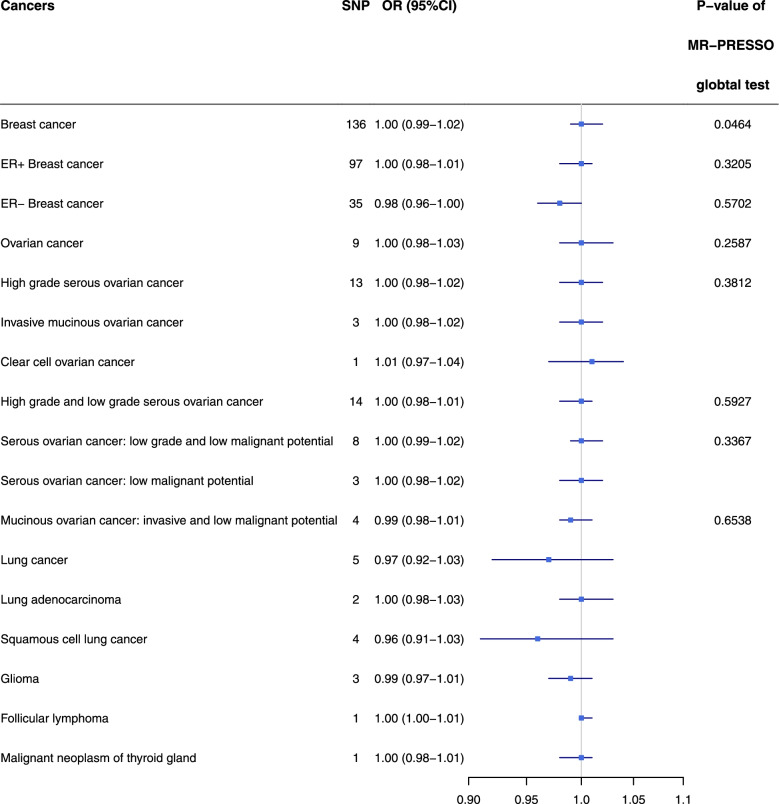
The causal estimates of different types of cancer on depression and the evaluation of their horizontal pleiotropy by MR-PRESSO. MR-PRESSO: Mendelian randomization-pleiotropy residual sum and outlier

In the dissection of causal effect of different types of cancer on depression, we firstly identified SNPs strongly associated with specific type of cancers as genetic instruments for exposure. However, the strongly associated SNPs were only identified within 17 types of cancer, among which we identified less than 5 associated SNPs within 9 types of cancer, and only 1 SNP within 3 types of cancer (Supplementary Table S9). Thus, we could not perform MR analysis for the other 9 types of cancer. The proportion of variance in different type of cancer explained by SNPs varies from 4.13% to 9.79% (Supplementary Table S13). The F-statistics of each type of cancer far exceeded 10 (Supplementary Table S13). Figure 3 demonstrates the results of primary MR analysis of the causal effect of a specific type of cancer on depression and the evaluation of pleiotropic effects. We also provided the scatter plots and funnel plots of each pair of association for better demonstration of causality and identification of heterogeneity. (Supplementary Figure S3-S4) In the primary MR analysis, we did not detect statistically significant causal effect of any type of cancer on depression by either IVW or weighted median method after the correction of heterogeneity. (Supplementary Table S9) Pleiotropy was detected in breast cancer with MR-Egger intercept test (*P* = 0.0464) and MR-PRESSO global test (*P* < 1.0 × 10^–4^) and lung cancer with MR-PRESSO global test (*P* = 0.0022), but no statistically significant causal effect could be identified after the correction of pleiotropic effect (Supplementary Table S10). No SNP was filtered for all types of cancer as exposure in Steiger filtering (Supplementary Table S12). The leave-one-out analysis showed that no single SNP disproportionately influenced the causal estimate (Supplementary Table S11, Supplementary Figures S31-S35). The power of each pair of association varied from 5 to 25% (Supplementary Table S13).

## Discussion

Depression has been reported to be associated with certain types of cancer in observational studies [5–8]. We performed the two-sample bi-directional MR with publicly available GWAS summary statistics to investigate the causal relationship between the genetically predicted depression and the risk of multiple types of cancers, including ovarian cancer, breast cancer, lung cancer, glioma, lymphoma, colorectal cancer, thyroid cancer, bladder cancer, and kidney cancer. We detected suggestive evidence for the causality of the genetically predicted depression on breast cancer (OR = 1.09, 95% CI: 1.03–1.15, *P* = 0.0022), but no evidence for reverse causality.

A previous observational study from the Taiwan National Health Insurance Research Database found that depression was independently associated with a 1.62-fold (95% CI: 1.12–2.34) overall increased risk of subsequent cancer during five-year follow-up [7]. Our study reveals that depression leaded to a higher risk of developing breast cancer (OR = 1.09, 95% CI: 1.03–1.15, *P* = 0.0022), and the causal estimate remained similar after correction for heterogeneity (OR = 1.09, 95% CI: 1.02–1.17, *P* = 0.0176) and pleiotropy (OR = 1.10, 95% CI: 1.03–1.16, *P* = 0.0072). The causal effect of depression on breast cancer estimated by weighted median method or estimated in the 3-cohort replication set (OR = 1.09, 95% CI: 1.03–1.15, *P* = 0.0037) also demonstrated consistency in direction and magnitude. Moreover, their causal relationship showed correct Steiger direction orientating from depression to breast cancer (*P* < 0.0001). These evidence suggest a robust causal effect of depression on breast cancer. A meta-analysis found that evidence from epidemiological studies was insufficient to support the causal effect of depression on breast cancer [34]. For lung cancer, the causal relationship between depression and lung cancer turned negative after Steiger filtering in our study, which is consistent with the results of the Baltimore Epidemiologic Catchment Area Study [8]. For ovarian cancer, a 19-year prospective cohort study did not find association between depression and ovarian cancer [35], while a pooled analysis of two large prospective cohort studies, Nurses’ Health Study I and II, demonstrated that depression diagnosed prior to cancer diagnosis was associated increased risk of ovarian cancer [5]. Our study did not identify the causal effect of depression on different types of ovarian cancer after Steiger filtering. The inconsistency between the results of MR and observational studies and the controversies among the results of observational studies might result from the confounding factors and bias in the real-world epidemiological studies. MR, an analogue for randomized controlled trials, is a more effective tool for causal inference as it is less likely to be affected by confounding [14].

Many mechanisms of depression’s carcinogenic effect have been proposed. Depression was deemed as a type of chronic stress. The perception of stress activates the hypothalamic–pituitary–adrenal axis and induces the release of stress hormone, such as cortisol which is involved in stress-related signaling as well as the regulation of cell growth and cell cycle [36]. Another possible pathway is the stress-induced impairment of immune function, in which the decreased cytotoxic T-cells and natural killer cells affect the immune surveillance [37]. In ovarian cancer, depression might also affect the repairment of post-ovulatory wounds, the accumulation of which might lead to the carcinogenesis of ovary surface epithelium [38]. In addition, depression may also result in the unhealthy behavior, including gluttony, anorexia, smoking, drinking, or lack of physical activity.

Reversely, depression is also a common comorbidity in cancer patients. Patients with cancer were reported to be at higher risk of depression [39]. The prevalence of depression varies among different types of cancer. Depression is more likely to be associated with pancreatic, breast, lung and oropharyngeal cancers [40]. A variety of factors were associated with increased risk of depression in patients with cancer, including poorly controlled pain, several types of medications, etc. However, the causal effect of different types of cancer on depression as well as their detailed mechanism have not been elucidated. In this MR analysis, no reverse causality was detected, but it does not prove the absence of the causal effect of cancer on depression. Among 17 types of cancer included in the MR analysis of reverse causality, all F-statistics were far more than 10, but the statistical power of the 11 types of cancer was only 5%, indicating the insufficiency of power to draw a reliable conclusion. Limited by the availability of full summary statistics, we only adopted the GWAS from a single consortium for each type of cancer respectively, rather than the pooled summary statistics from the meta-analysis. The prevalence of cancer is far less than depression, and the sample size of different types of cancer are also less than the sample size of depression. Thus, the number of adopted SNPs is extremely low for most types of cancer. 14 types of cancer adopted less than 20 strongly associated SNPs as genetic instruments for exposure in MR analysis.

There were several strengths in our study. First, as depression could not be randomly assigned as an intervention to different groups, observational study is the only feasible method for the clinical study of depression. In this study, we utilized randomly allocated genetic variants as instrumental variables, which can avoid the reverse causality and the bias introduced by cofounders. Second, we have covered up to 26 types of cancer for MR analysis, to provide a systematic investigation of the causal relationships. Third, the data source of each pair of exposure and outcome is non-overlapping, ensuring the basic requirements of the two-sample MR analysis. Fourth, the F-statistics for each exposure all exceeded 10, which indicates no weak instrument bias. Finally, in addition to the conventional fixed-effect IVW method, we used random-effect IVW, MR-Egger and MR-PRESSO method to correct for heterogeneity and horizontal pleiotropy respectively, and we also used weighted median method as a sensitivity analysis to ensure the validity of the conclusions drawn on the causality.

Some limitations of this study should also be pointed out. First, limited by the availability of the full summary statistics of depression GWAS from 23AndMe Inc, we only utilized the full summary statistics of depression GWAS from PGC and UK Biobank, so the results of our study might be underpowered. Second, as two-sample MR requires that both samples come from the same population, our study only includes participants of European ancestry, so the generalization of these results to other populations requires further studies. Third, the number of strongly associated SNPs selected in the MR analysis of reverse causality is relatively small. The null result could be due to low power and insufficient SNPs, which restrict our capability of drawing conclusions of true causal relationship. Fourth, the inclusion criteria of depression participants in PGC cohort and UK Biobank cohort differs greatly. The PGC cohort used clinical diagnosis international consensus criteria (DSM-IV, ICD-9, or ICD-10), while UK Biobank cohort used self-diagnosis. The inconsistency of inclusion criteria might affect the associations between SNPs and depression, but Howard et al. detected strong genetic correlations between them, which suggested that similar underlying genetic architecture was captured by different studies [16]. Finally, the causal relationship might be confounded by hidden population structure [41]. Although the populations in our study were all European ancestry, within-population structures were not considered. Concerns were also raised about the sexual disparities between the population of depression and breast cancer, as the between-sex difference in the prevalence of depression was reported in some epidemiological studies [42]. The Major Depressive Disorder Working Group of the PGC investigated between-sex genetic heterogeneity in major depressive disorder with GWAS summary statistics from multiple cohorts including PGC, Kaiser Permanente GERA, UK Biobank, and the Danish iPSYCH studies, but did not detect convincing evidence for between-sexes genetic differences [43]. As a complex multifactorial disease, the between-sex difference in the prevalence of depression might result from biological, psychological, environmental, or other factors [44]. Given the controversies, a gender-stratified MR analysis might provide more confirmatory evidence. However, generalized analytic method has not been established considering the challenging and complex nature of population structure, and stratification correction might lead to overcorrection [45]. In addition, these publicly available data did not provide sufficient information for further analysis of impact from population structure.

## Conclusions

In summary, the results of this study suggested the potential causal effect of genetically predicted depression on breast cancer among 26 types of cancer, while causal effect of depression on other types of cancer and reverse causality were not identified. It indicates the importance of mental health in the prevention and treatment of cancer. However, our findings were partly consistent with existing observational studies. Thus, to better illustrate the causal relationships between depression and different types of cancer, further verification in more large-scale prospective studies as well as deeper mechanistic studies are warranted.

## Supplementary Information

**Additional file 1.**

**Additional file 2.**

## Data Availability

GWAS summary statistics for depression can be downloaded from Howard’s meta-analysis [16]. GWAS summary statistics for breast cancer can be downloaded from the BCAC consortium website (http://bcac.ccge.medschl.cam.ac.uk/bcacdata/). GWAS summary statistics for ovarian cancer can be downloaded from the OCAC consortium website (http://ocac.ccge.medschl.cam.ac.uk/data-projects/). GWAS summary statistics for lung cancer from the ILCCO consortium is publicly available. GWAS summary statistics for glioma can be downloaded through the National Center for Biotechnology Information database of Genotypes and Phenotypes (dbGaP) website (https://www.ncbi.nlm.nih.gov/projects/gap/cgi-bin/study.cgi?study_id=phs000652.v1.p1). GWAS summary statistics for pancreatic cancer can be downloaded through the dbGaP website (https://www.ncbi.nlm.nih.gov/projects/gap/cgi-bin/study.cgi?study_id=phs000206.v5.p3). GWAS summary statistics for lymphoma, colorectal cancer, thyroid cancer, bladder cancer, and kidney cancer excluding renal pelvis can be downloaded from the FinnGen consortium website (https://finngen.gitbook.io/documentation/data-download). GWAS summary statistics for the above-mentioned cancers can also be accessed from MR-Base database (http://app.mrbase.org/).

## Abbreviations

BCAC: Breast cancer association consortium
GliomaScan: Cohort-based genome-wide association study of glioma
HR: Hazard ratio
ILCCO: International lung cancer consortium
INSIDE: Instrument strength independent of direct effect
IVW: Inverse-variance weighted
LD: Linkage disequilibrium
MR: Mendelian randomization
MR-PRESSO: Mendelian randomization pleiotropy residual sum and outlier
OCAC: Ovarian cancer association consortium
OR: Odds ratio
PanScan: Pancreatic cancer cohort consortium
PGC: Psychiatric genomics consortium
RR: Relative risk
WHO: World health organization
