# Supplementary material for: Causal relationship between genetically predicted depression and cancer risk: a two-sample bi-directional mendelian randomization
Source: BMC Cancer. 2022 Mar 31;22:353. doi: 10.1186/s12885-022-09457-9 (PMC8973550; doi:10.1186/s12885-022-09457-9)
Supplement: Supplementary file 1 — Additional file 1. [file 12885_2022_9457_MOESM1_ESM.pdf]

Supplementary Figures

Figure S1: Scatter plot of the causal effect of depression on different types of cancer, with the slope of each line corresponding to estimated causal effect per method 4

Figure S2: Funnel plot of the causal effect of depression on different types of cancer...... 6

Figure S3: Scatter plot of the causal effect of different types of cancer on depression, with the slope of each line corresponding to estimated causal effect per method 8

Figure S4: Funnel plot of the causal effect of different types of cancer on depression...... 9

Figure S5: Leave-one-out inverse-variance weighted mendelian randomization analyses of depression on Breast cancer 10

Figure S6: Leave-one-out inverse-variance weighted mendelian randomization analyses of depression on ER+ Breast cancer 11

Figure S7: Leave-one-out inverse-variance weighted mendelian randomization analyses of depression on ER- Breast cancer 12

Figure S8: Leave-one-out inverse-variance weighted mendelian randomization analyses of depression on Ovarian cancer... 13

Figure S9: Leave-one-out inverse-variance weighted mendelian randomization analyses of depression on High grade serous ovarian cancer... 14

Figure S10: Leave-one-out inverse-variance weighted mendelian randomization analyses of depression on Low grade serous ovarian cancer 15

Figure S11: Leave-one-out inverse-variance weighted mendelian randomization analyses of depression on Invasive mucinous ovarian cancer.. 16

Figure S12: Leave-one-out inverse-variance weighted mendelian randomization analyses of depression on Clear cell ovarian cancer 17

Figure S13: Leave-one-out inverse-variance weighted mendelian randomization analyses of depression on Endometrioid ovarian cancer.. 18

Figure S14: Leave-one-out inverse-variance weighted mendelian randomization analyses of depression on High grade and low grade serous ovarian cancer 19

Figure S15: Leave-one-out inverse-variance weighted mendelian randomization analyses of depression on Serous ovarian cancer: low grade and low malignant potential 20

Figure S16: Leave-one-out inverse-variance weighted mendelian randomization analyses of depression on Serous ovarian cancer: low malignant potential 21

Figure S17: Leave-one-out inverse-variance weighted mendelian randomization analyses of depression on Mucinous ovarian cancer: invasive and low malignant potential 22

Figure S18: Leave-one-out inverse-variance weighted mendelian randomization analyses of depression on Lung adenocarcinoma 23

Figure S19: Leave-one-out inverse-variance weighted mendelian randomization analyses of depression on Lung cancer 24

Figure S20: Leave-one-out inverse-variance weighted mendelian randomization analyses of depression on Squamous cell lung cancer 25

Figure S21: Leave-one-out inverse-variance weighted mendelian randomization analyses of depression on Glioma 26

Figure S22: Leave-one-out inverse-variance weighted mendelian randomization analyses of depression on Pancreatic cancer 27

Figure S23: Leave-one-out inverse-variance weighted mendelian randomization analyses of depression on Colon adenocarcinoma 28

Figure S24: Leave-one-out inverse-variance weighted mendelian randomization analyses of depression on Colorectal cancer 29

Figure S25: Leave-one-out inverse-variance weighted mendelian randomization analyses of depression on Follicular lymphoma 30

Figure S26: Leave-one-out inverse-variance weighted mendelian randomization analyses of depression on Non-follicular lymphoma 31

Figure S27: Leave-one-out inverse-variance weighted mendelian randomization analyses of depression on Other and unspecified types of non-Hodgkin lymphoma..................... 32

Figure S28: Leave-one-out inverse-variance weighted mendelian randomization analyses of depression on Malignant neoplasm of bladder 33

Figure S29: Leave-one-out inverse-variance weighted mendelian randomization analyses of depression on Malignant neoplasm of thyroid gland.. 34

Figure S30: Leave-one-out inverse-variance weighted mendelian randomization analyses of depression on Malignant neoplasm of kidney, except renal pelvis 35

Figure S31: Leave-one-out inverse-variance weighted mendelian randomization analyses of Breast cancer on depression 36

Figure S32: Leave-one-out inverse-variance weighted mendelian randomization analyses of ER+ Breast cancer on depression 37

Figure S33: Leave-one-out inverse-variance weighted mendelian randomization analyses of ER- Breast cancer on depression 38

Figure S34: Leave-one-out inverse-variance weighted mendelian randomization analyses of different types of ovarian cancer on depression 39

Figure S35: Leave-one-out inverse-variance weighted mendelian randomization analyses of different types of cancer on depression 40

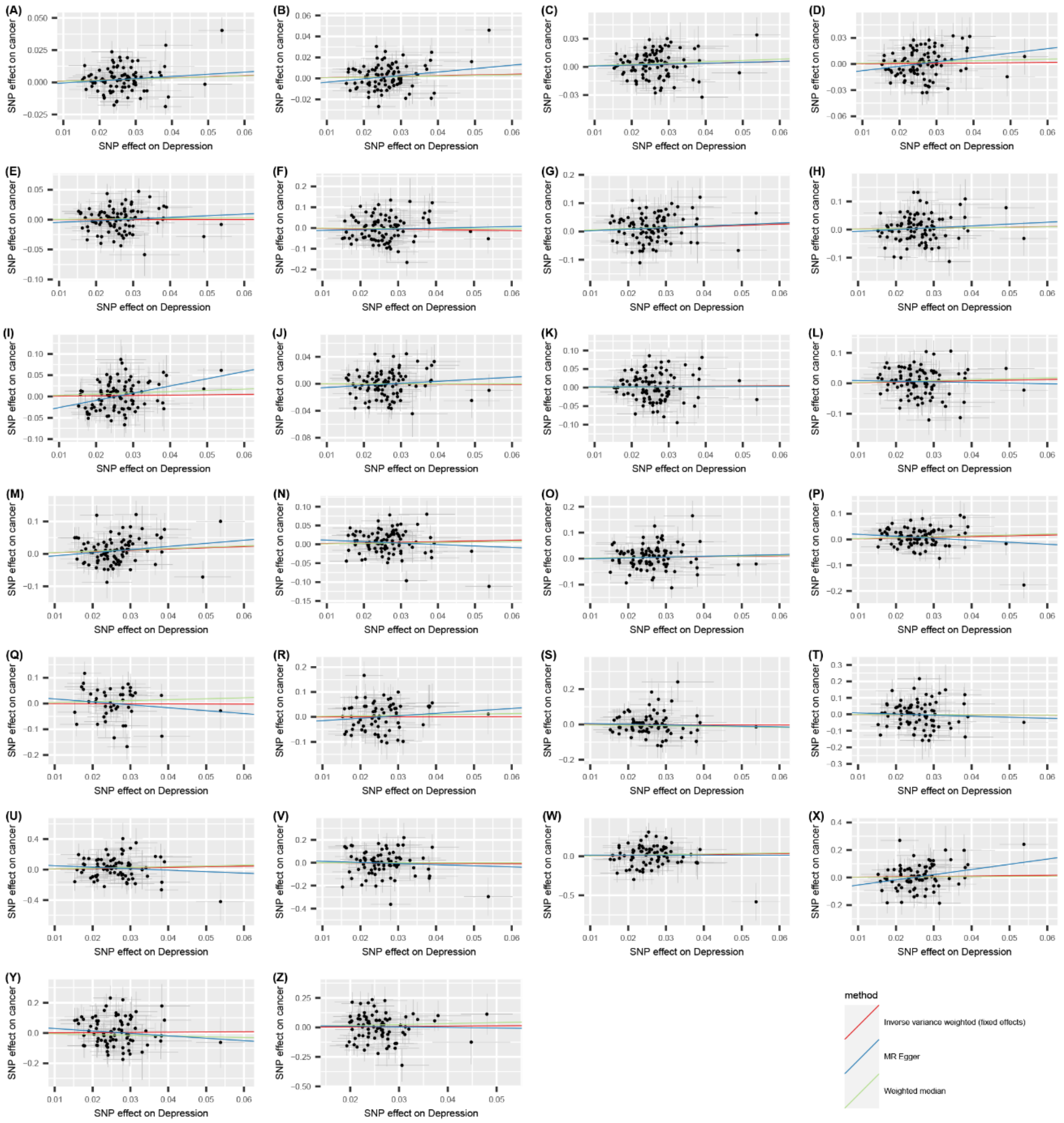

Figure S1: Scatter plot of the causal effect of depression on different types of cancer, with the slope of each line corresponding to estimated causal effect per method: (A) Breast cancer; (B) ER+ Breast cancer; (C) ER- Breast cancer; (D) Ovarian cancer; (E) High grade serous ovarian cancer; (F) Low grade serous ovarian cancer; (G) Invasive mucinous ovarian cancer; (H) Clear cell ovarian cancer; (I) Endometrioid ovarian cancer; (J) High grade and low grade serous ovarian cancer; (K) Serous ovarian cancer: low grade and low malignant potential; (L) Serous ovarian cancer: low malignant potential; (M) Mucinous ovarian cancer: invasive and low malignant potential; (N) Lung cancer; (O) Lung adenocarcinoma; (P) Squamous cell lung cancer; (Q) Glioma; (R) Pancreatic

cancer; (S) Colorectal cancer; (T) Colon adenocarcinoma; (U) Follicular lymphoma; (V) Non-follicular lymphoma; (W) Other and unspecified types of non-Hodgkin lymphoma; (X) Malignant neoplasm of bladder; (Y) Malignant neoplasm of thyroid gland; (Z) Malignant neoplasm of kidney, except renal pelvis.

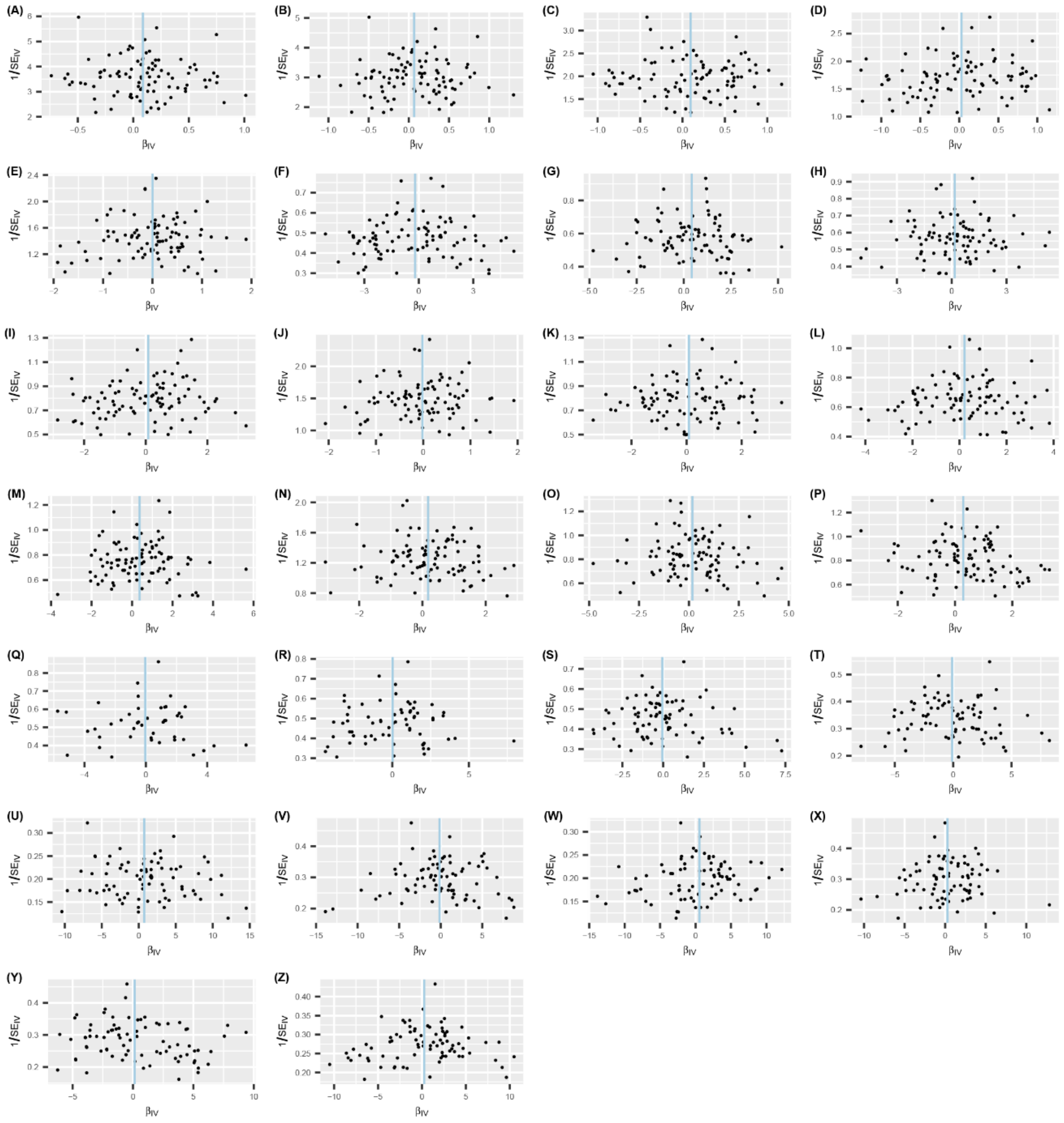

Figure S2: Funnel plot of the causal effect of depression on different types of cancer: (A) Breast cancer; (B) ER+ Breast cancer; (C) ER- Breast cancer; (D) Ovarian cancer; (E) High grade serous ovarian cancer; (F) Low grade serous ovarian cancer; (G) Invasive mucinous ovarian cancer; (H) Clear cell ovarian cancer; (I) Endometrioid ovarian cancer; (J) High grade and low grade serous ovarian cancer; (K) Serous ovarian cancer: low grade and low malignant potential; (L) Serous ovarian cancer: low malignant potential; (M) Mucinous ovarian cancer: invasive and low malignant potential; (N) Lung cancer; (O) Lung adenocarcinoma; (P) Squamous cell lung cancer; (Q) Glioma; (R) Pancreatic cancer; (S) Colorectal cancer; (T) Colon adenocarcinoma; (U) Follicular lymphoma; (V)

Non-follicular lymphoma; (W) Other and unspecified types of non-Hodgkin lymphoma; (X) Malignant neoplasm of bladder; (Y) Malignant neoplasm of thyroid gland; (Z) Malignant neoplasm of kidney, except renal pelvis.

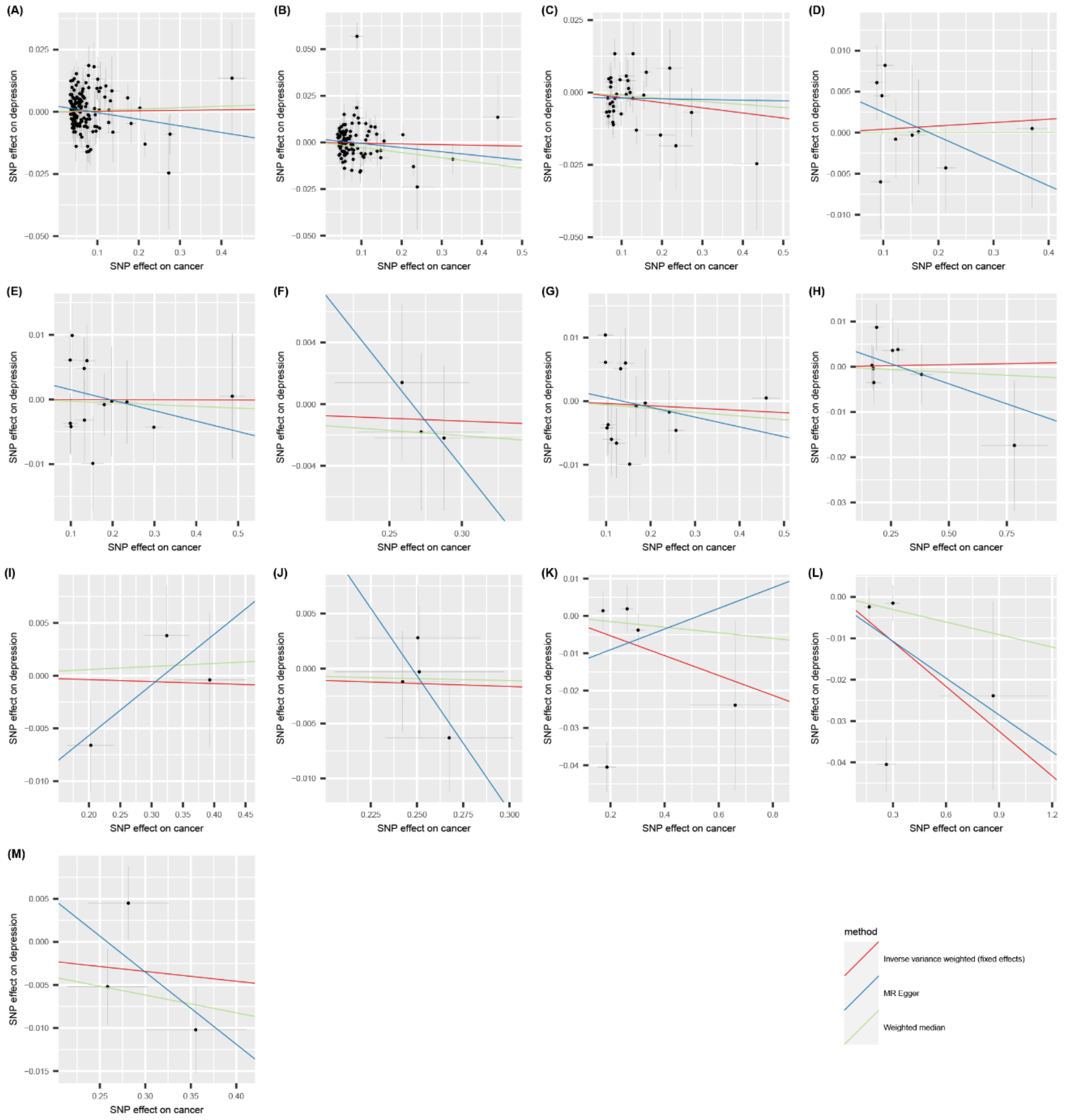

Figure S3: Scatter plot of the causal effect of different types of cancer on depression, with the slope of each line corresponding to estimated causal effect per method: (A) Breast cancer; (B) ER+ Breast cancer; (C) ER- Breast cancer; (D) Ovarian cancer; (E) High grade serous ovarian cancer; (F) Invasive mucinous ovarian cancer; (G) High grade and low grade serous ovarian cancer; (H) Serous ovarian cancer: low grade and low malignant potential; (I) Serous ovarian cancer: low malignant potential; (J) Mucinous ovarian cancer: invasive and low malignant potential; (K) Lung cancer; (L) Squamous cell lung cancer; (M) Glioma.

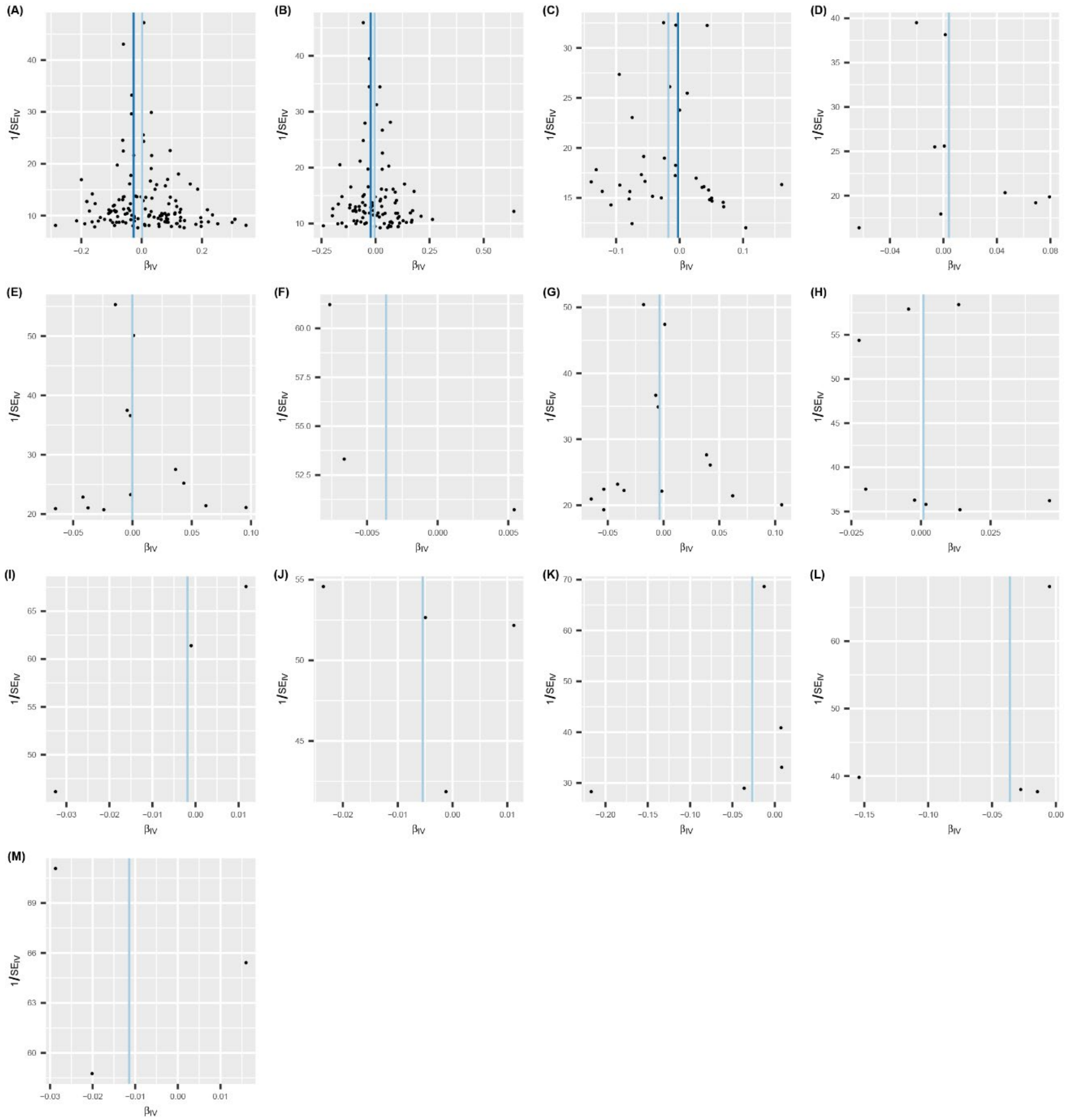

Figure S4: Funnel plot of the causal effect of different types of cancer on depression: (A) Breast cancer; (B) ER+ Breast cancer; (C) ER- Breast cancer; (D) Ovarian cancer; (E) High grade serous ovarian cancer; (F) Invasive mucinous ovarian cancer; (G) High grade and low grade serous ovarian cancer; (H) Serous ovarian cancer: low grade and low malignant potential; (I) Serous ovarian cancer: low malignant potential; (J) Mucinous ovarian cancer: invasive and low malignant potential; (K) Lung cancer; (L) Squamous cell lung cancer; (M) Glioma.

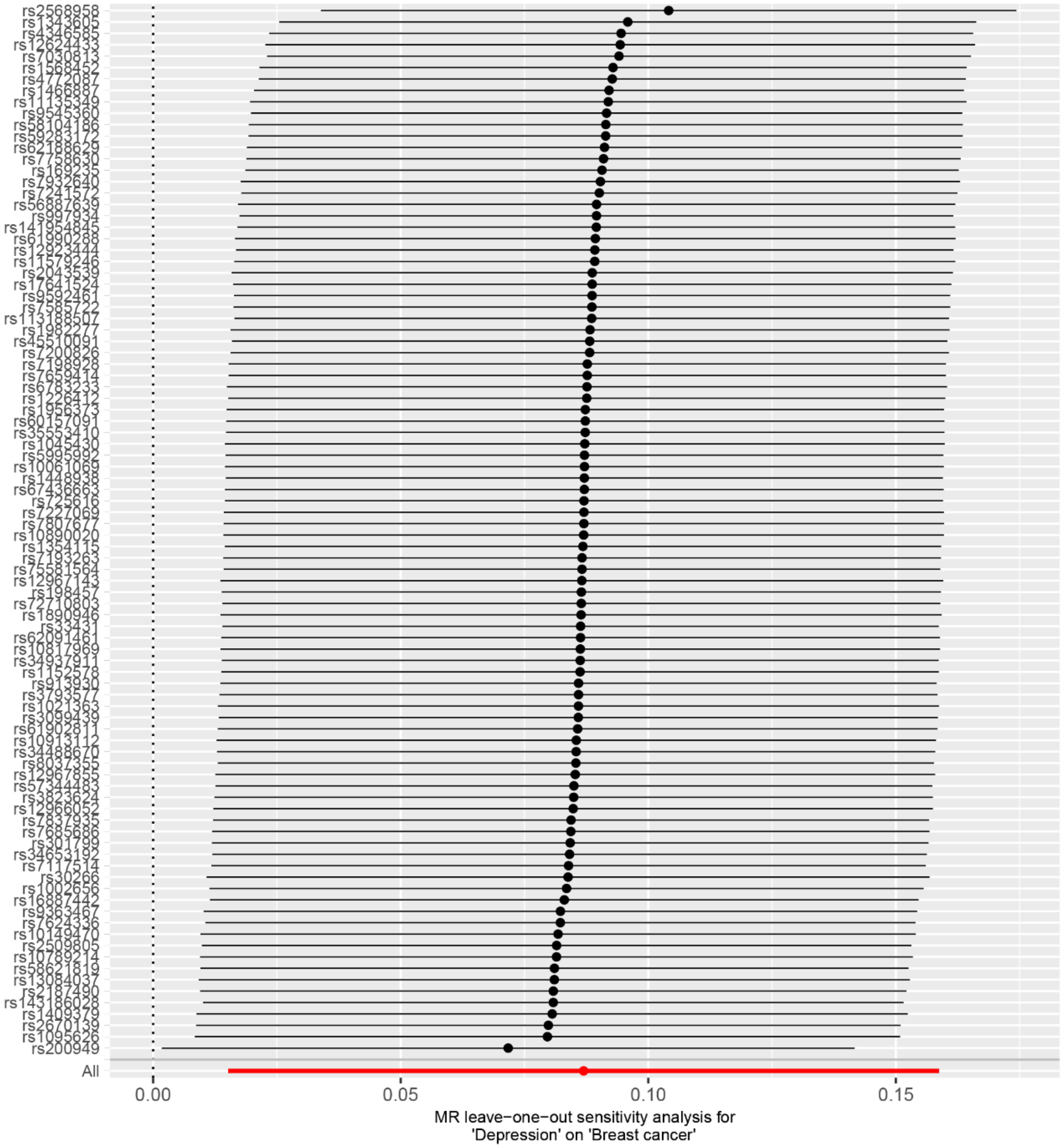

Figure S5: Leave-one-out inverse-variance weighted mendelian randomization analyses of depression on Breast cancer

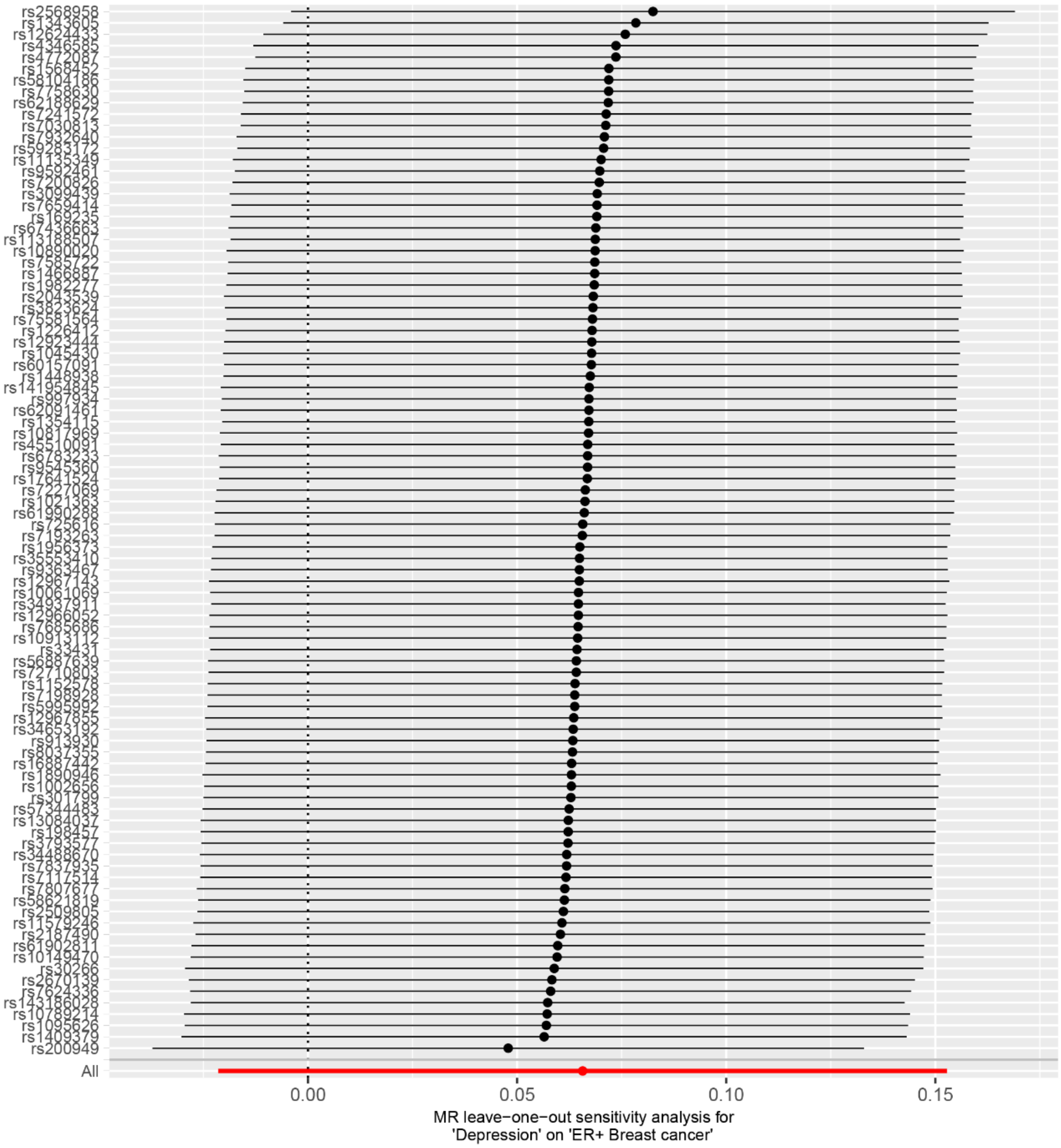

Figure S6: Leave-one-out inverse-variance weighted mendelian randomization analyses of depression on ER+ Breast cancer

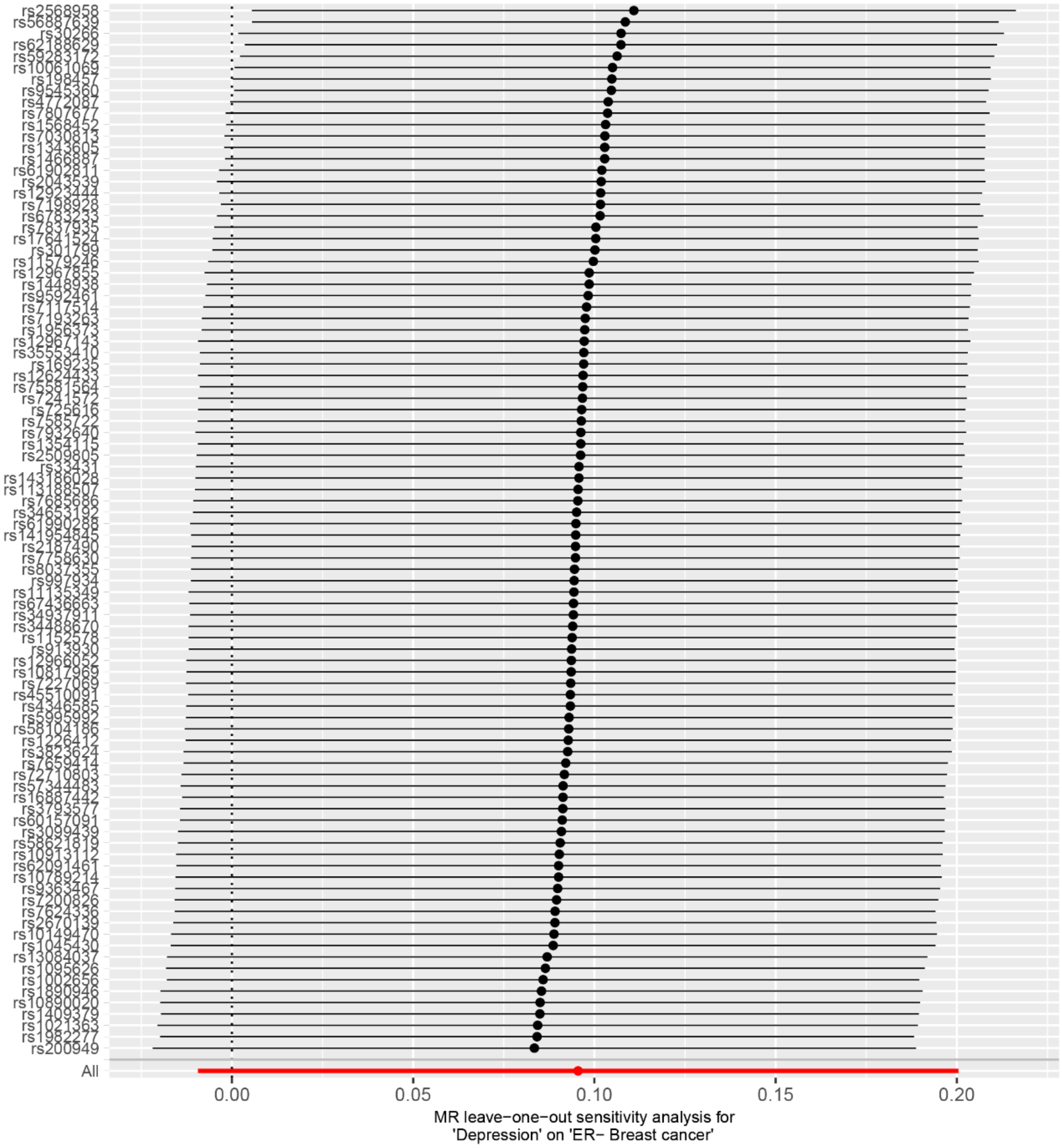

Figure S7: Leave-one-out inverse-variance weighted mendelian randomization analyses of depression on ER- Breast cancer

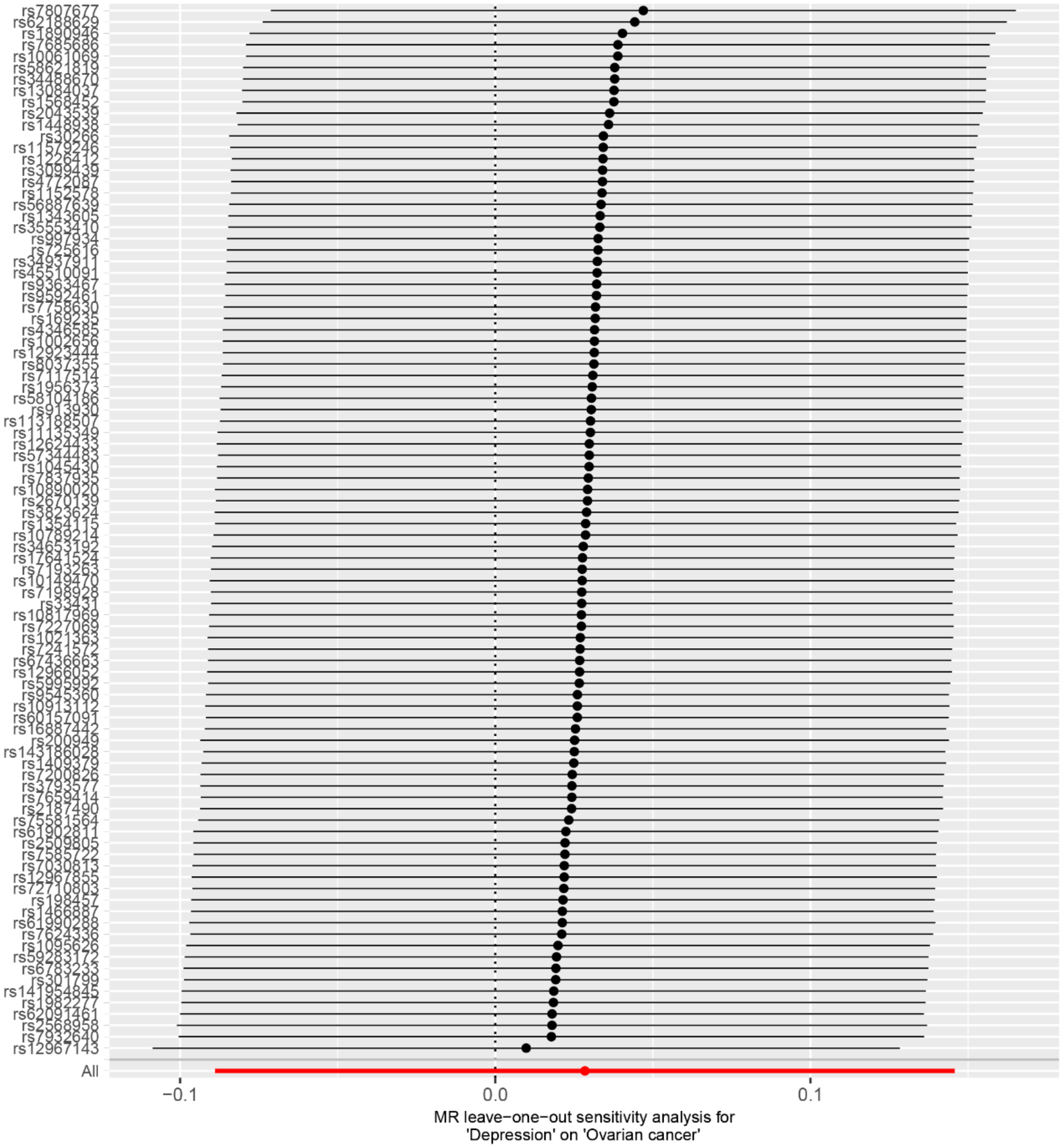

Figure S8: Leave-one-out inverse-variance weighted mendelian randomization analyses of depression on Ovarian cancer

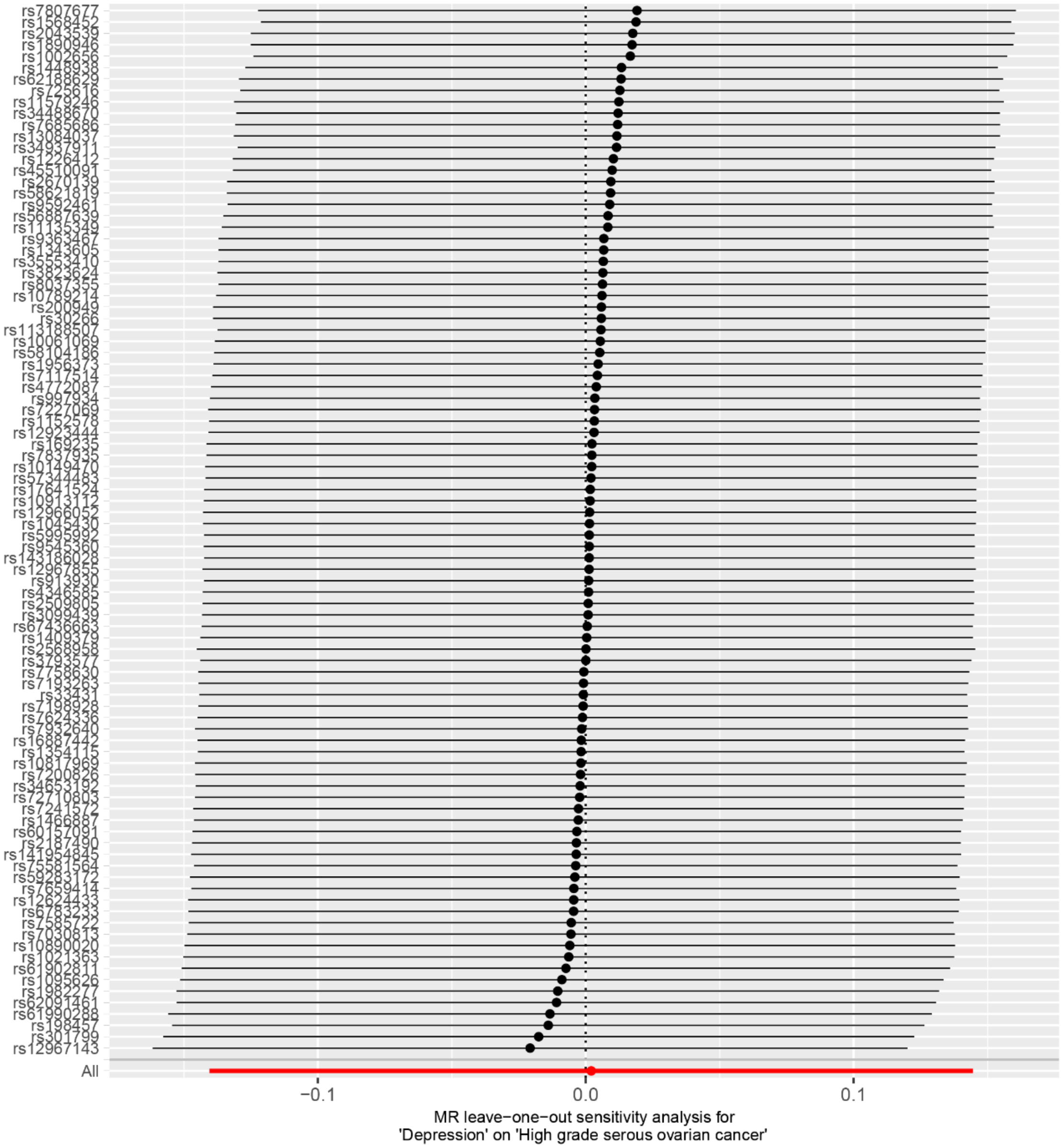

Figure S9: Leave-one-out inverse-variance weighted mendelian randomization analyses of depression on High grade serous ovarian cancer

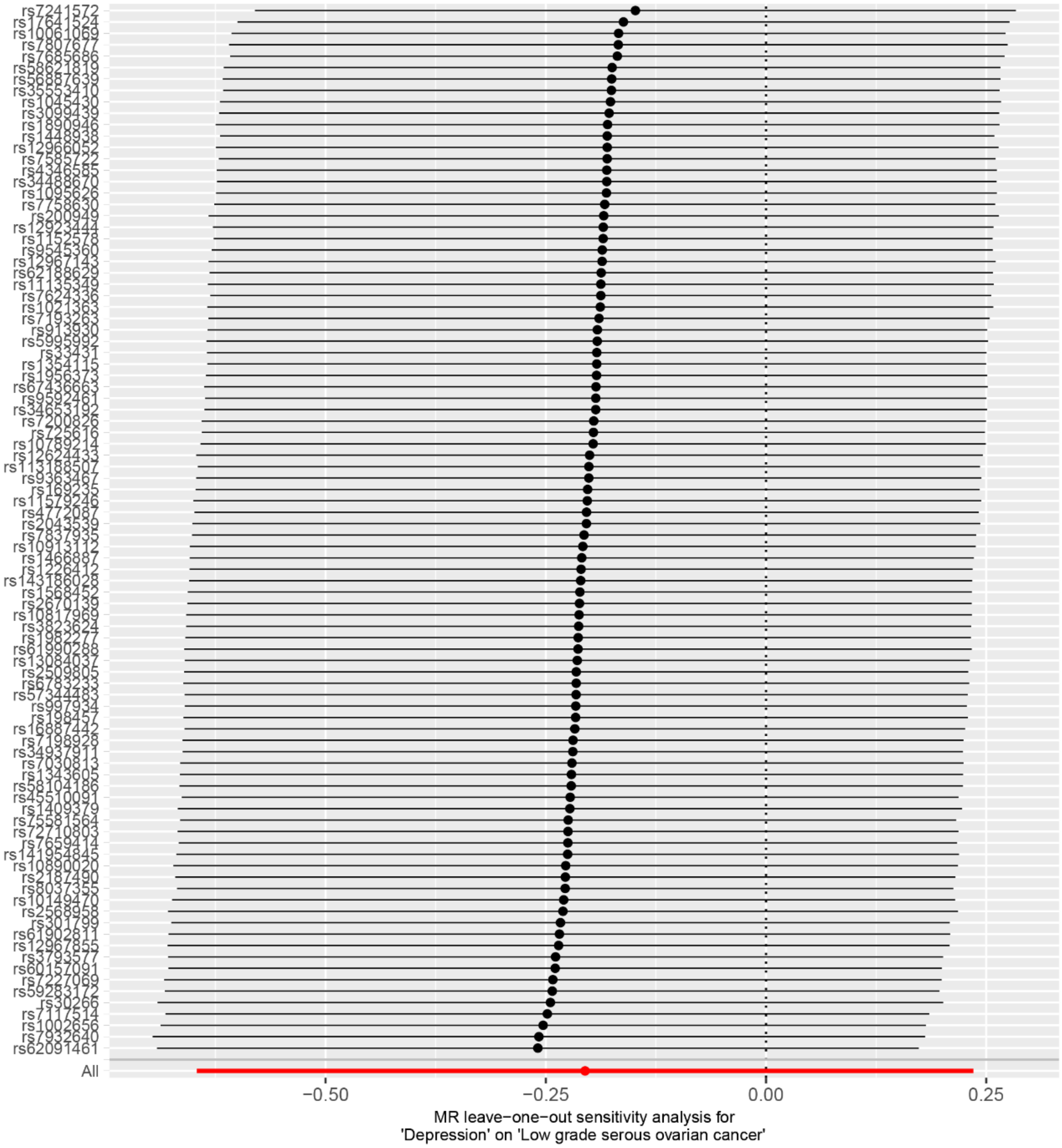

Figure S10: Leave-one-out inverse-variance weighted mendelian randomization analyses of depression on Low grade serous ovarian cancer

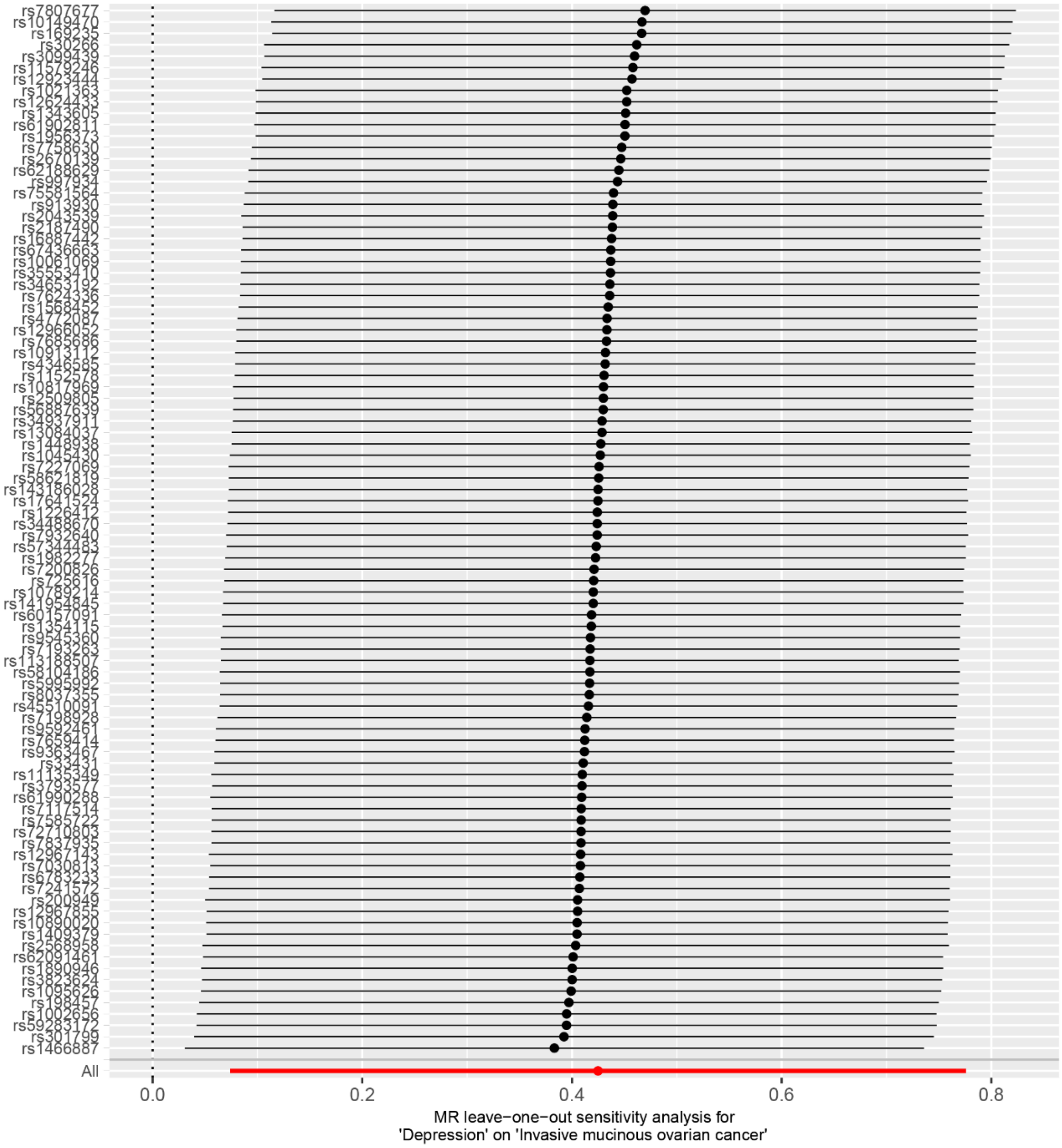

Figure S11: Leave-one-out inverse-variance weighted mendelian randomization analyses of depression on Invasive mucinous ovarian cancer

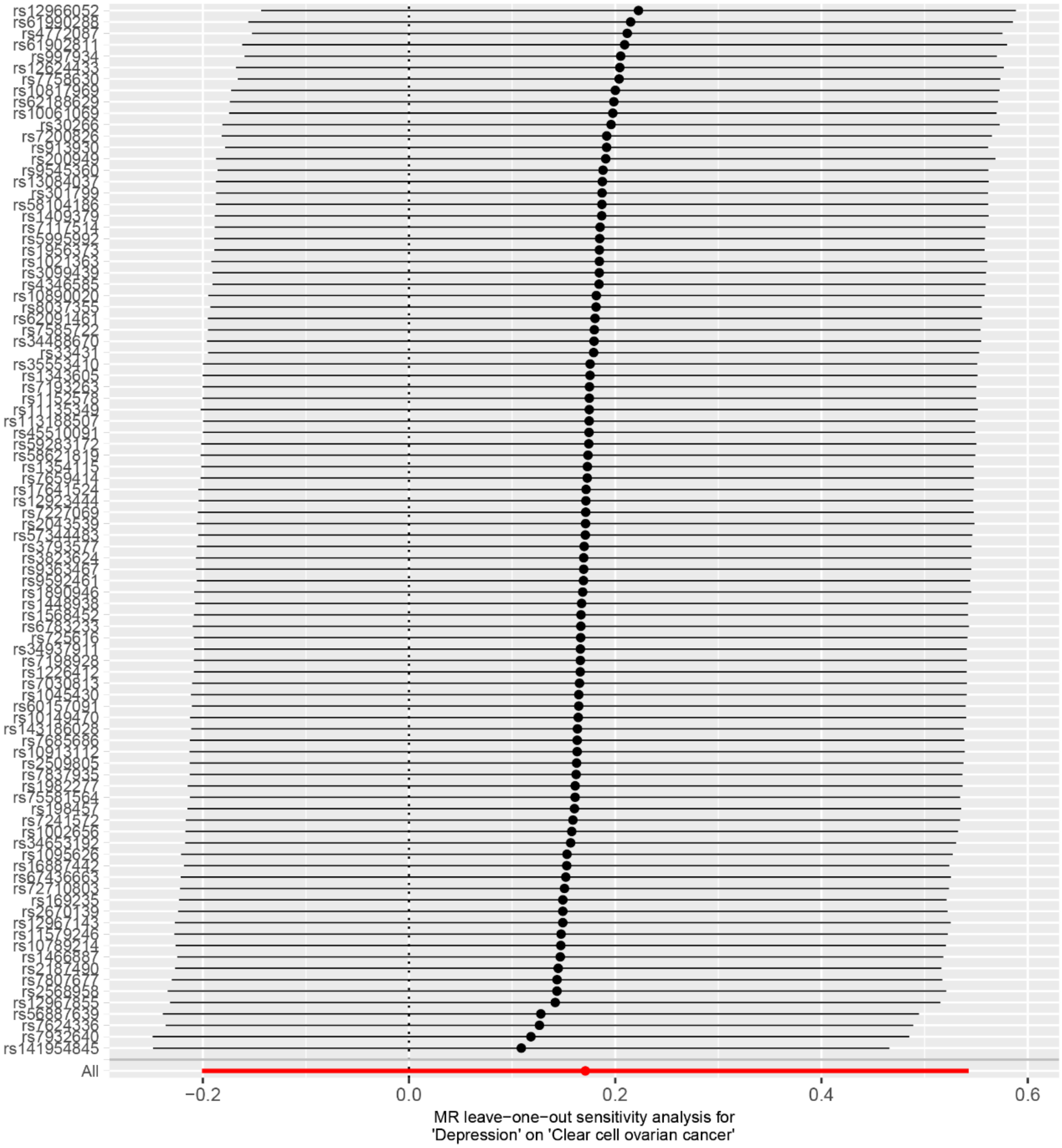

Figure S12: Leave-one-out inverse-variance weighted mendelian randomization analyses of depression on Clear cell ovarian cancer

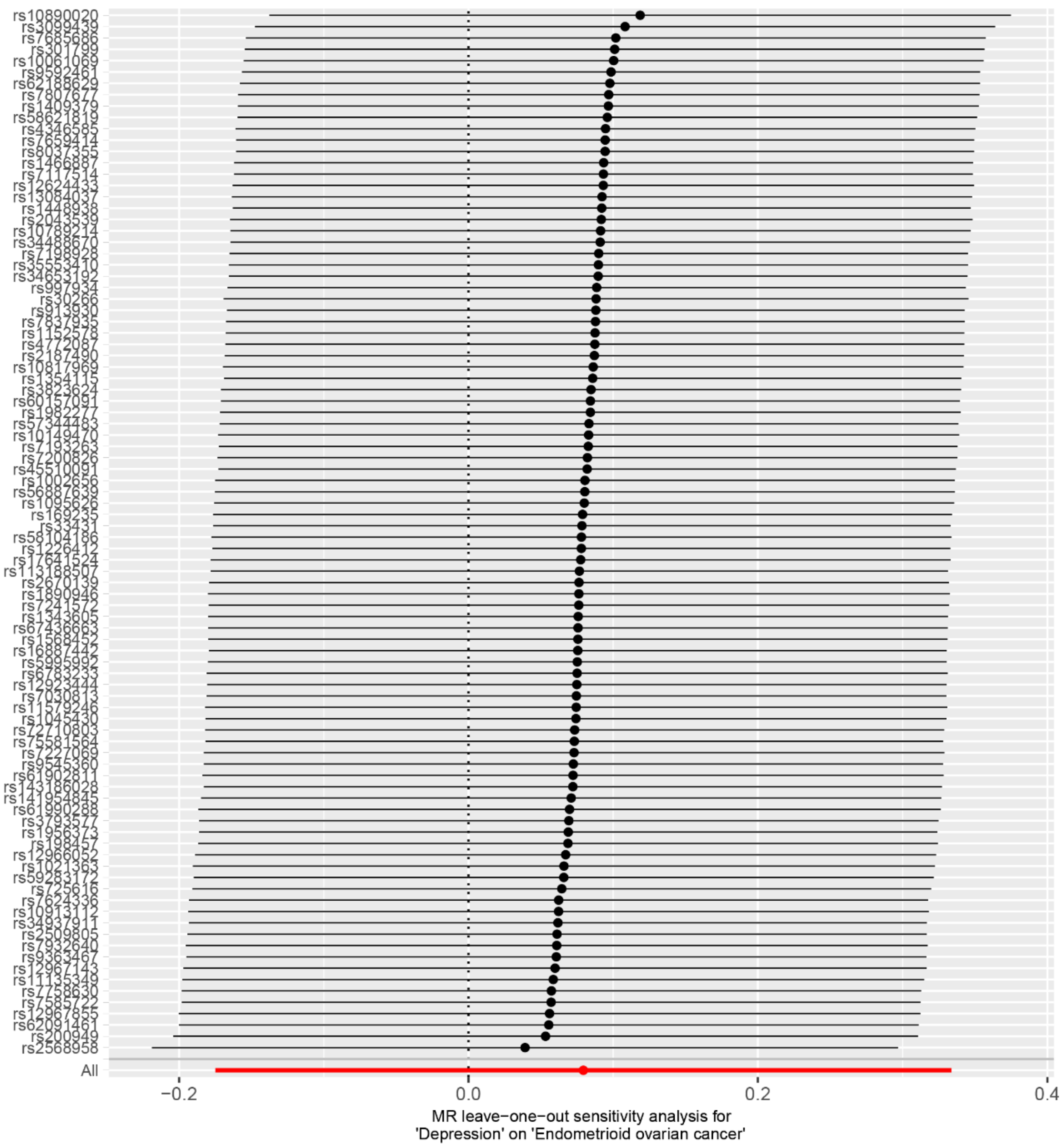

Figure S13: Leave-one-out inverse-variance weighted mendelian randomization analyses of depression on Endometrioid ovarian cancer

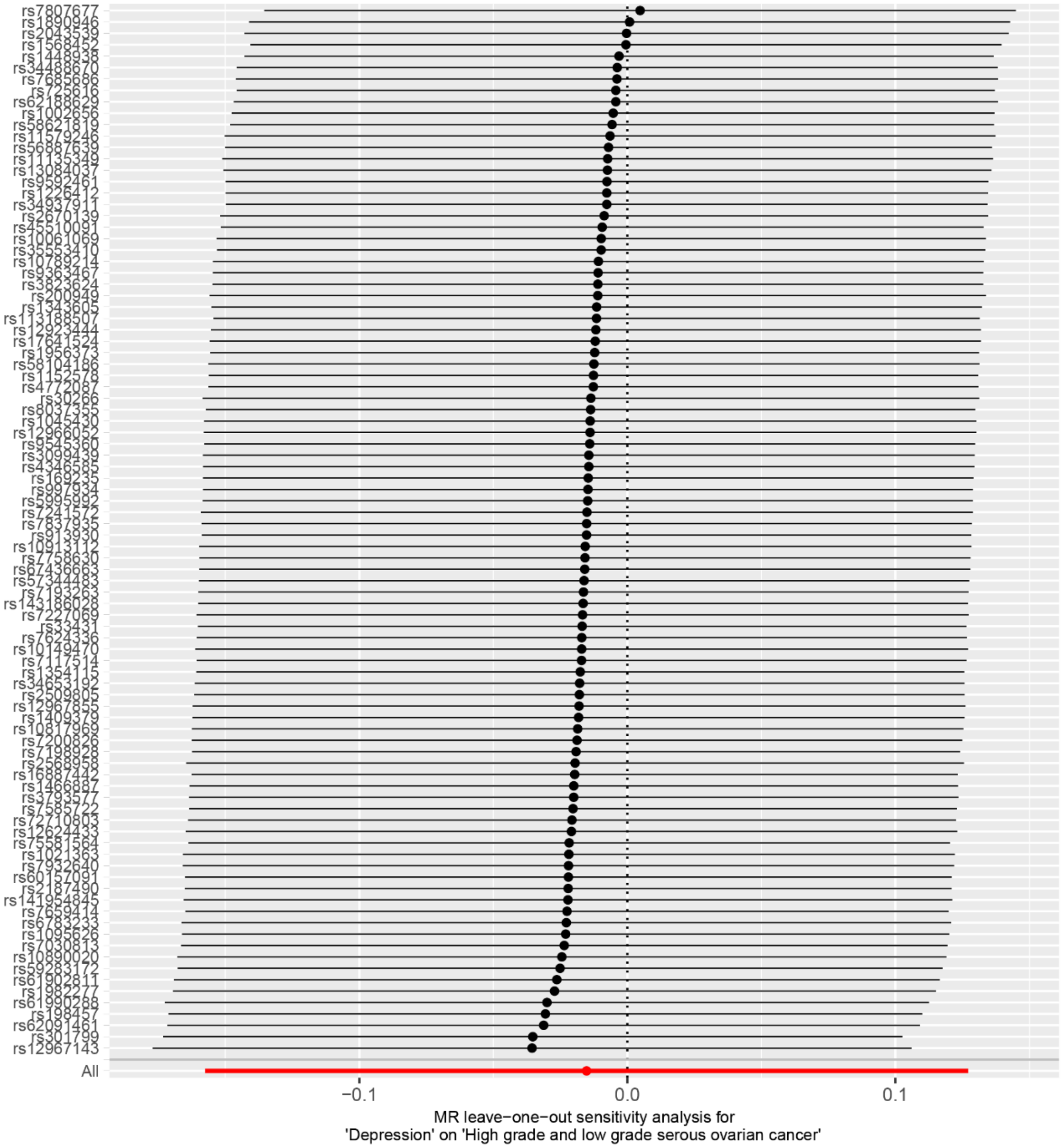

Figure S14: Leave-one-out inverse-variance weighted mendelian randomization analyses of depression on High grade and low grade serous ovarian cancer

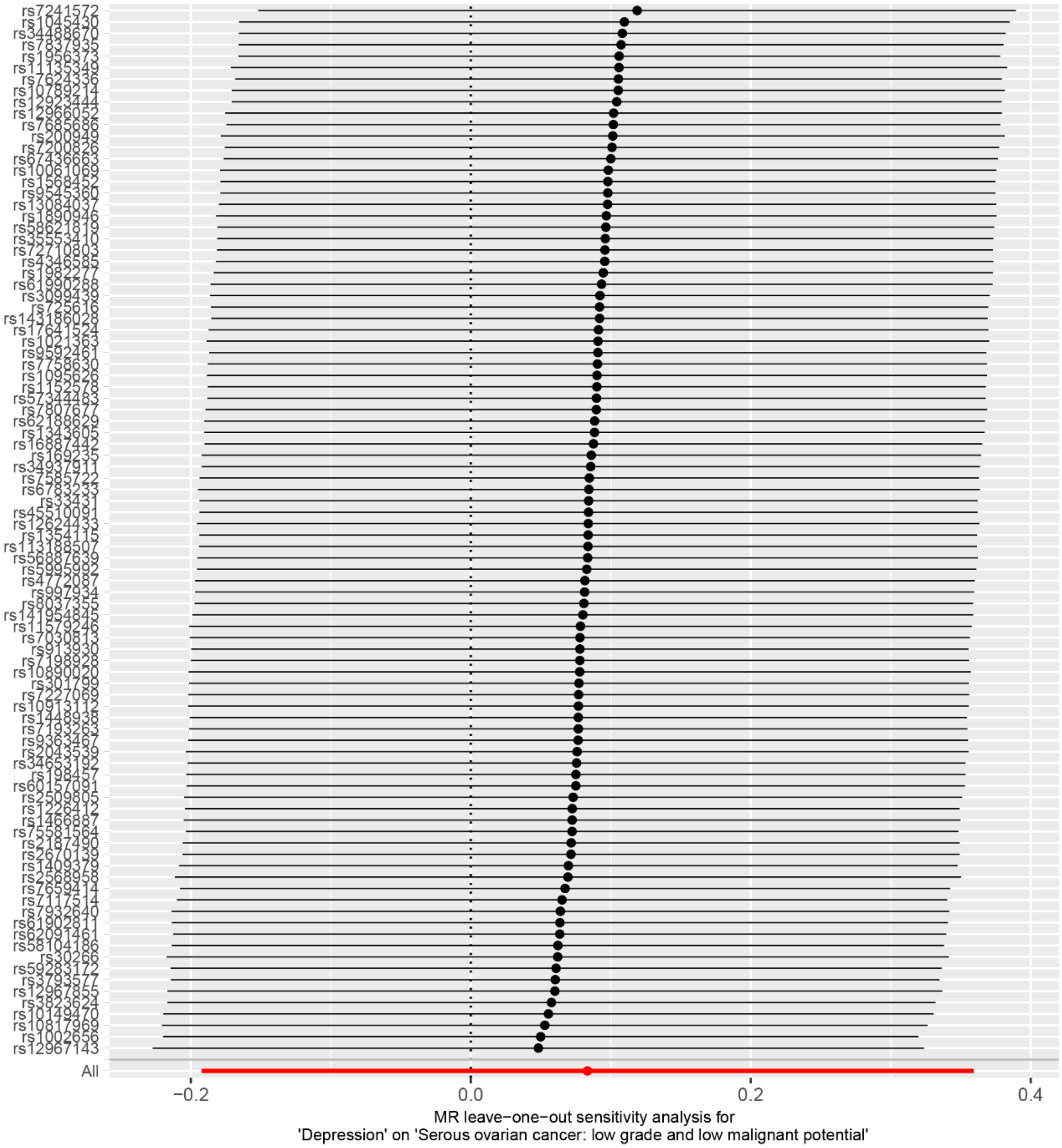

Figure S15: Leave-one-out inverse-variance weighted mendelian randomization analyses of depression on Serous ovarian cancer: low grade and low malignant potential

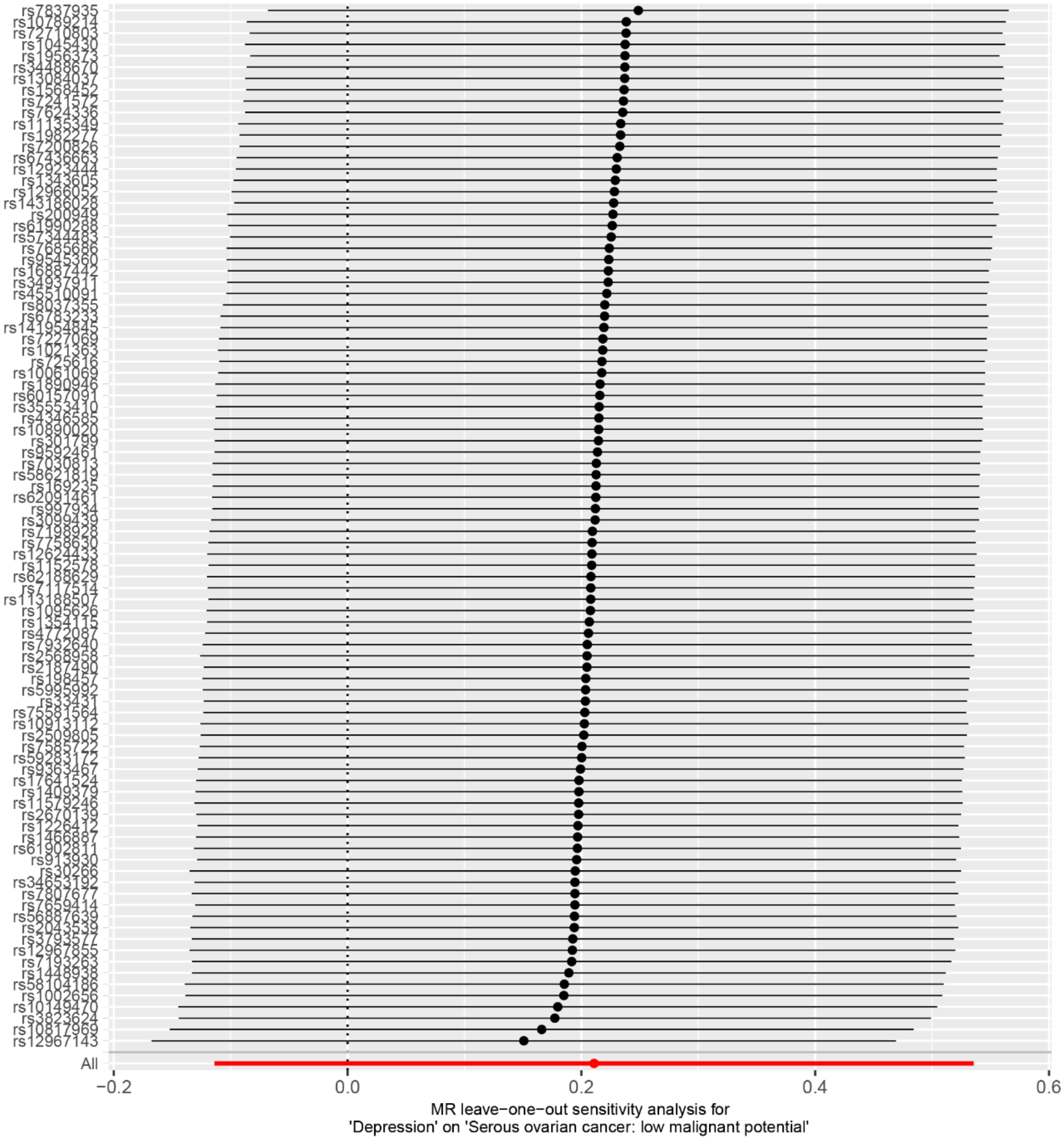

Figure S16: Leave-one-out inverse-variance weighted mendelian randomization analyses of depression on Serous ovarian cancer: low malignant potential

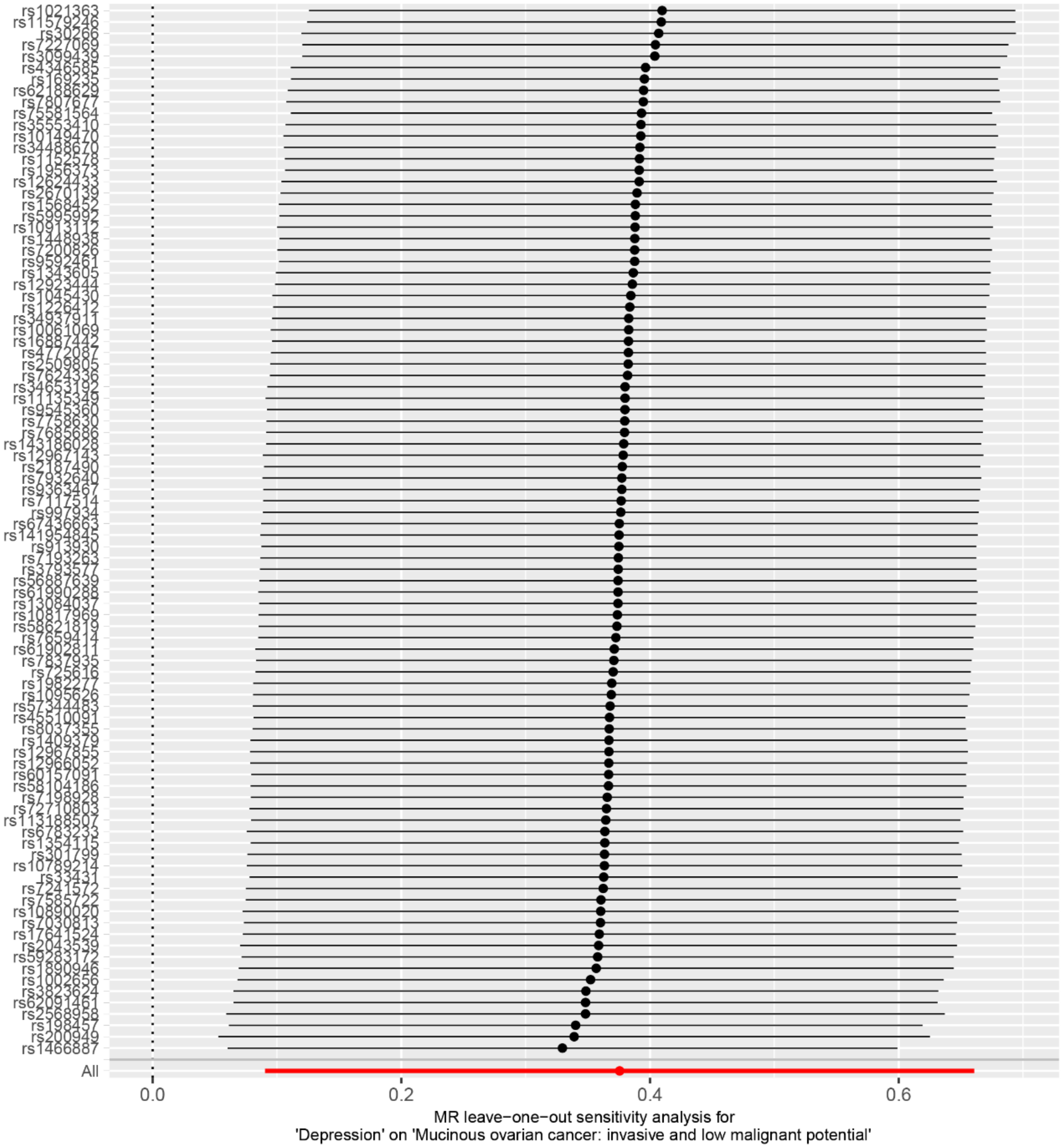

Figure S17: Leave-one-out inverse-variance weighted mendelian randomization analyses of depression on Mucinous ovarian cancer: invasive and low malignant potential

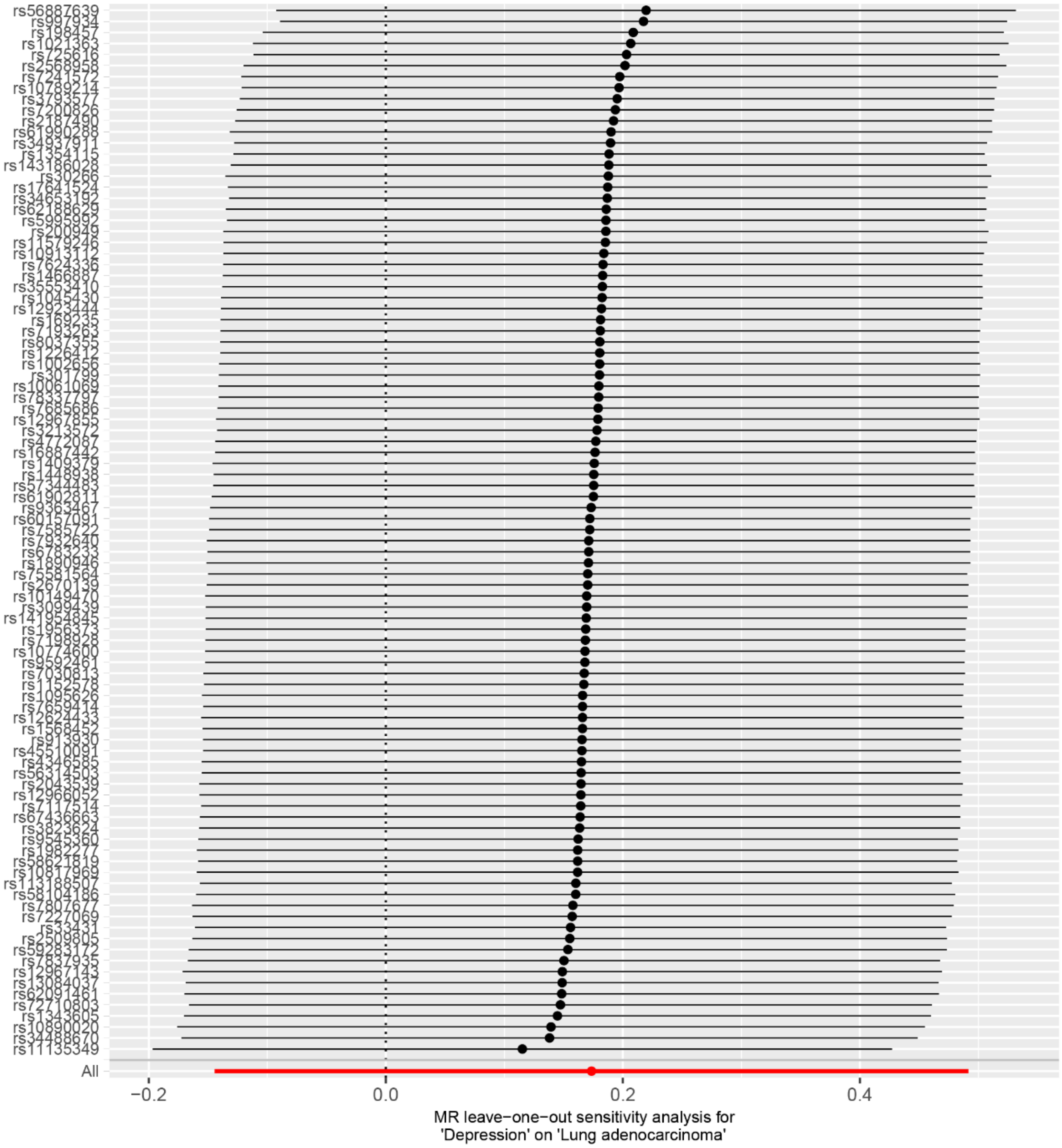

Figure S18: Leave-one-out inverse-variance weighted mendelian randomization analyses of depression on Lung adenocarcinoma

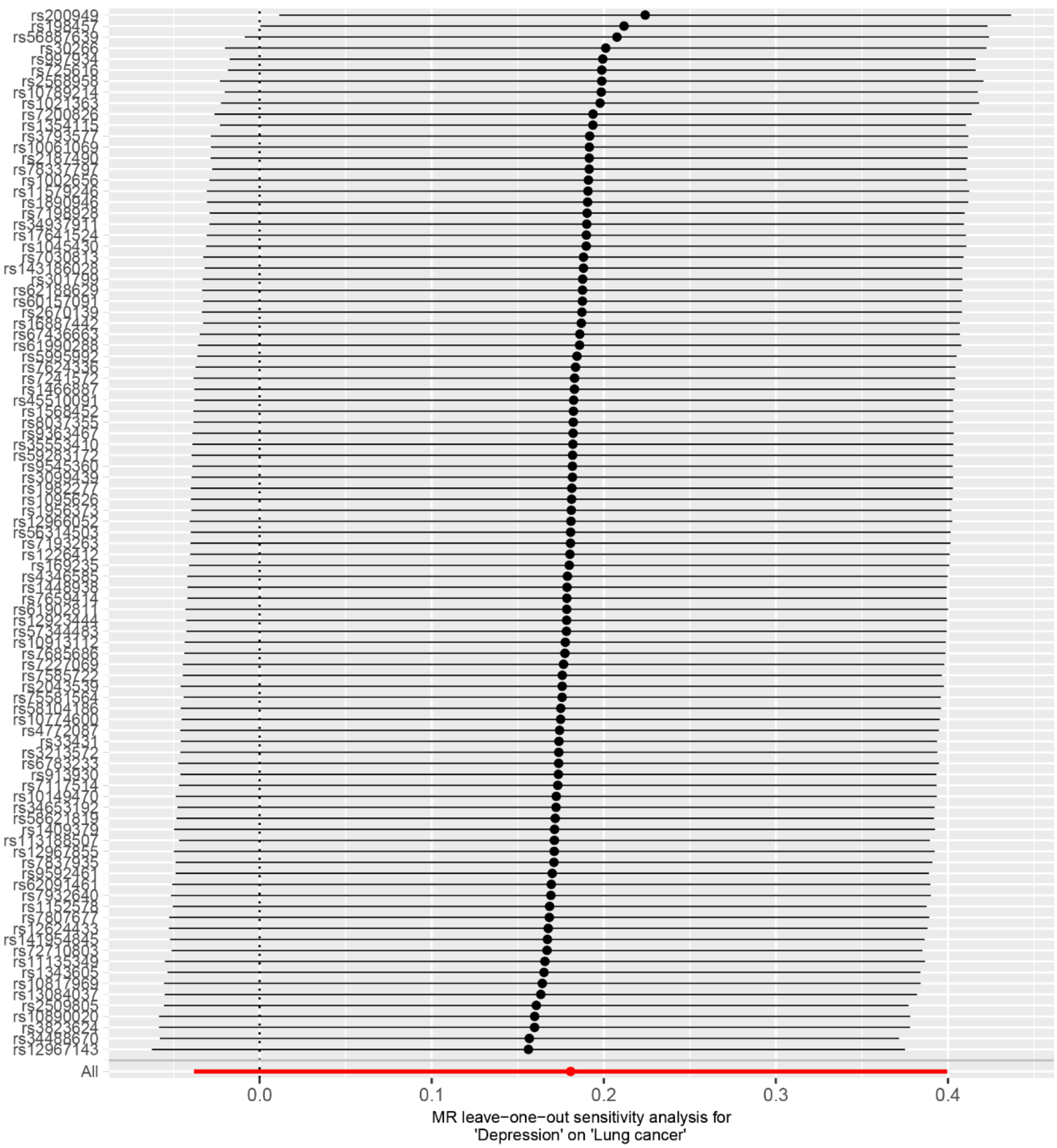

Figure S19: Leave-one-out inverse-variance weighted mendelian randomization analyses of depression on Lung cancer

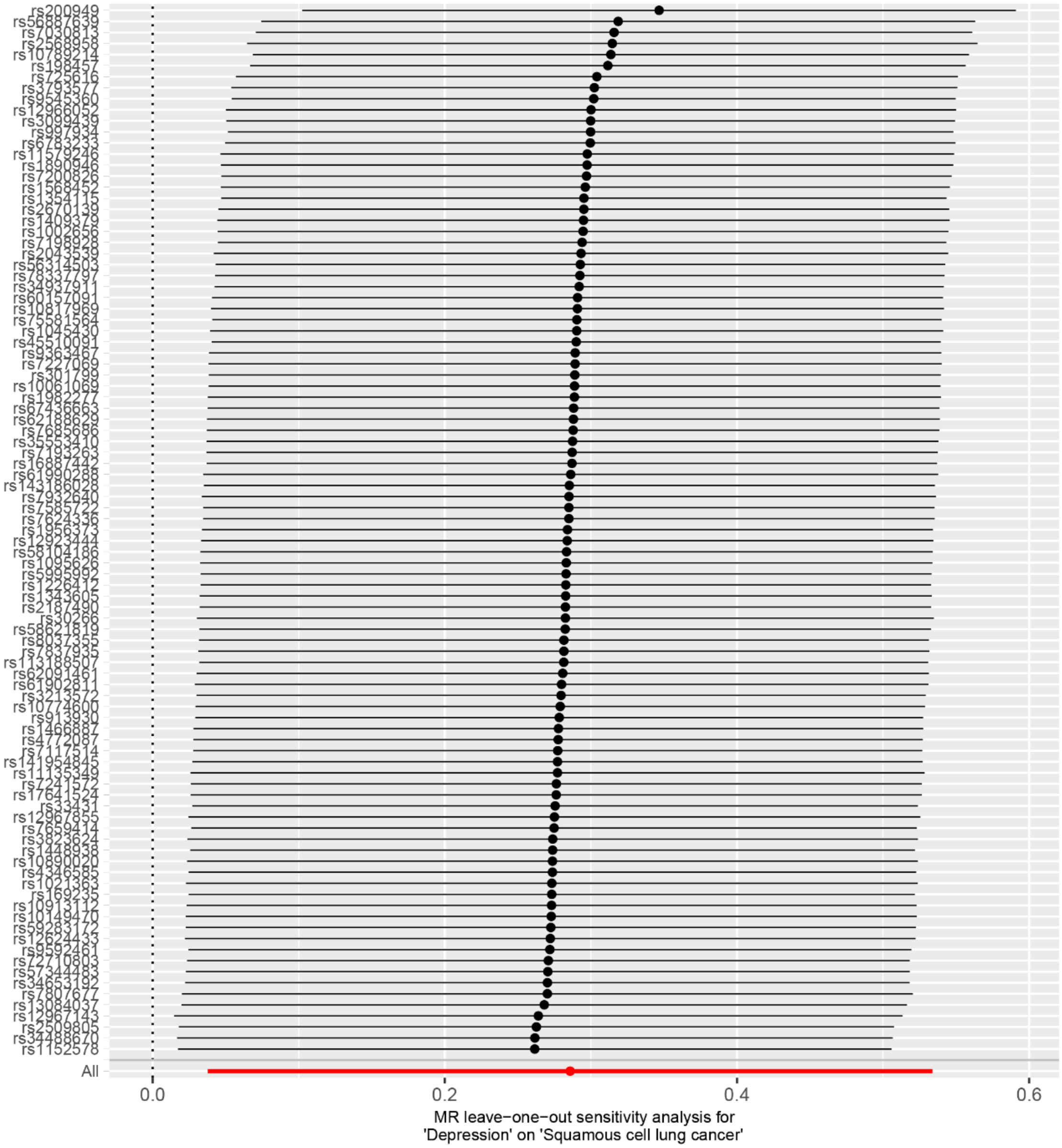

Figure S20: Leave-one-out inverse-variance weighted mendelian randomization analyses of depression on Squamous cell lung cancer

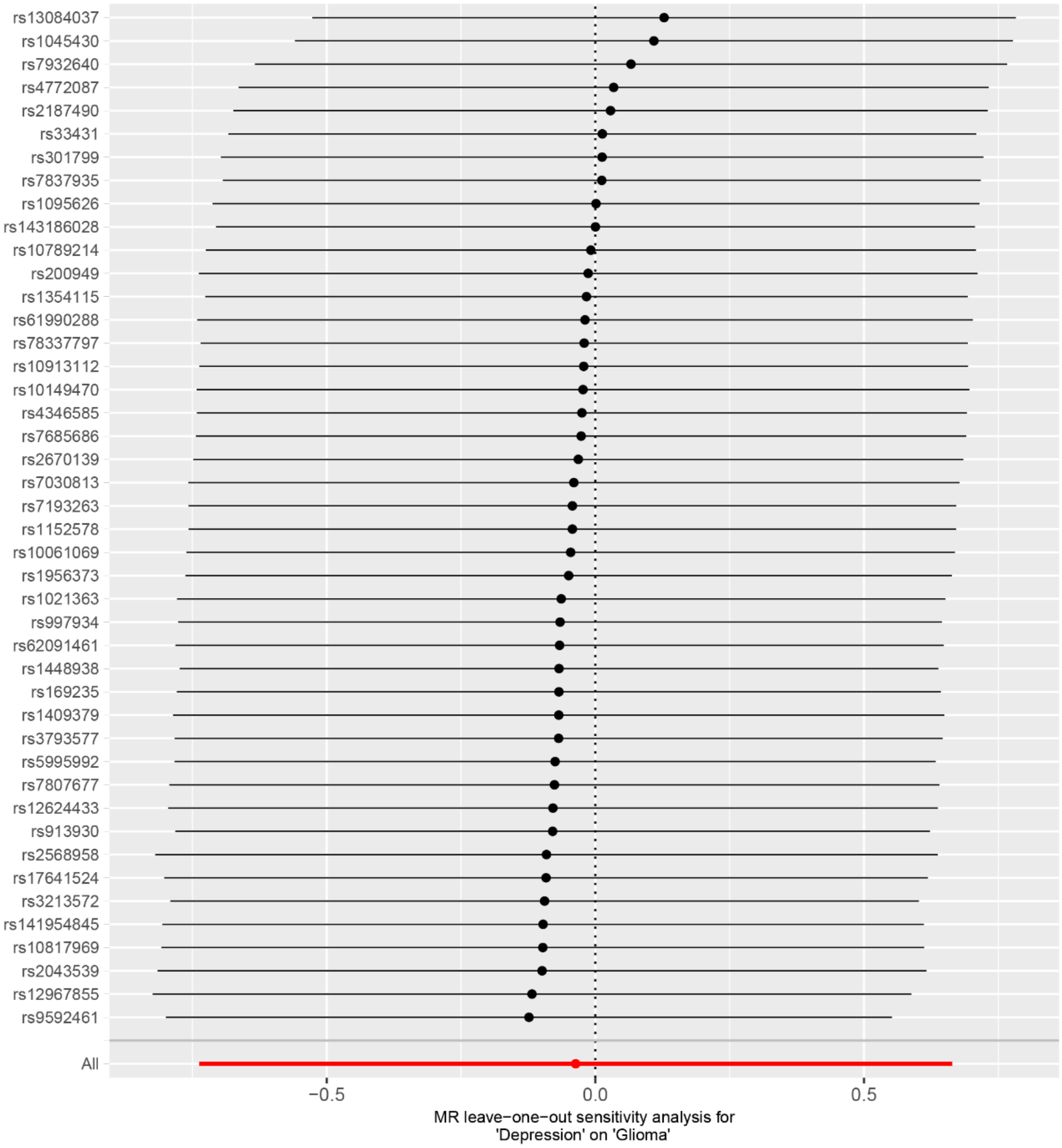

Figure S21: Leave-one-out inverse-variance weighted mendelian randomization analyses of depression on Glioma

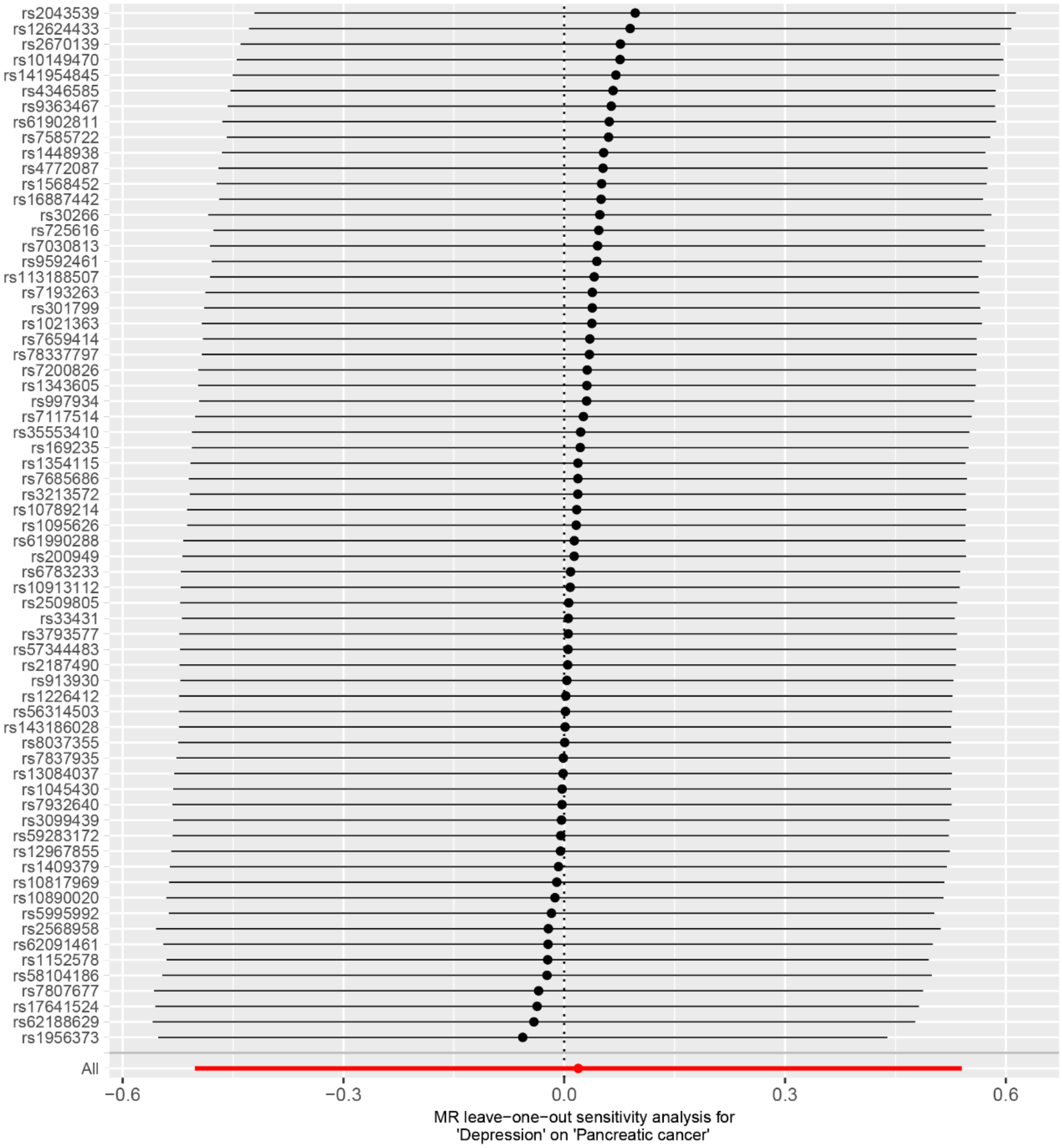

Figure S22: Leave-one-out inverse-variance weighted mendelian randomization analyses of depression on Pancreatic cancer

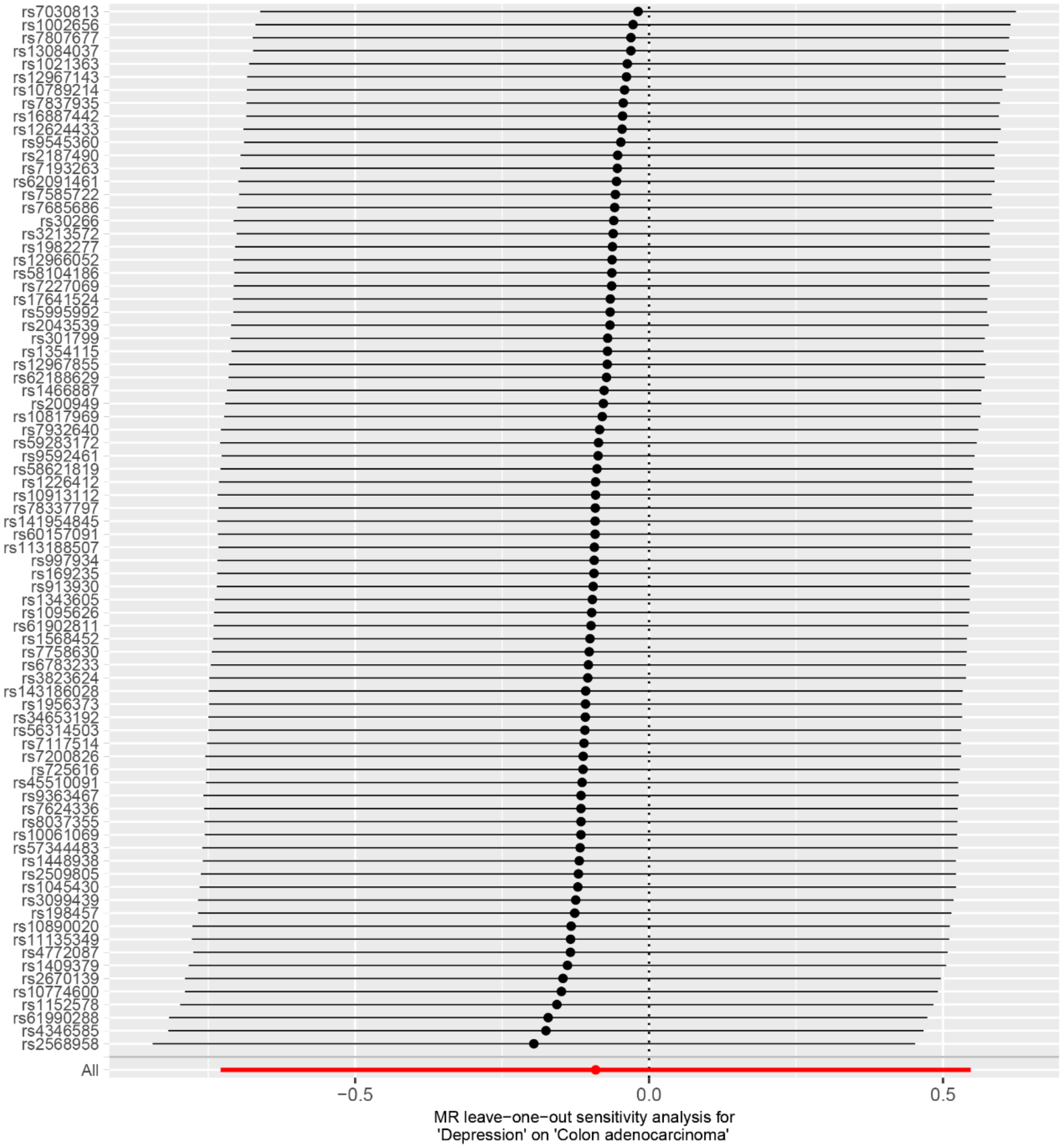

Figure S23: Leave-one-out inverse-variance weighted mendelian randomization analyses of depression on Colon adenocarcinoma

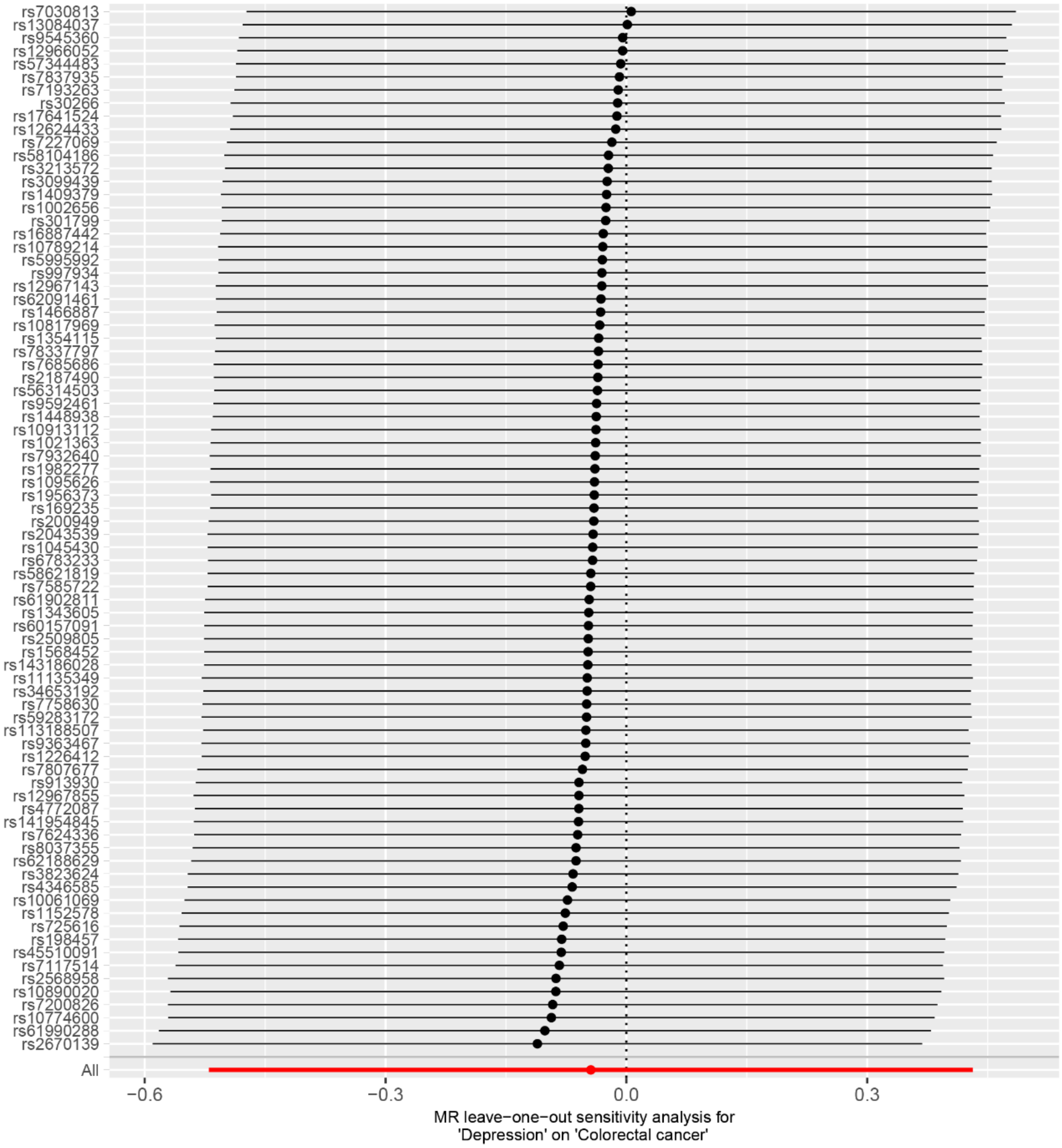

Figure S24: Leave-one-out inverse-variance weighted mendelian randomization analyses of depression on Colorectal cancer

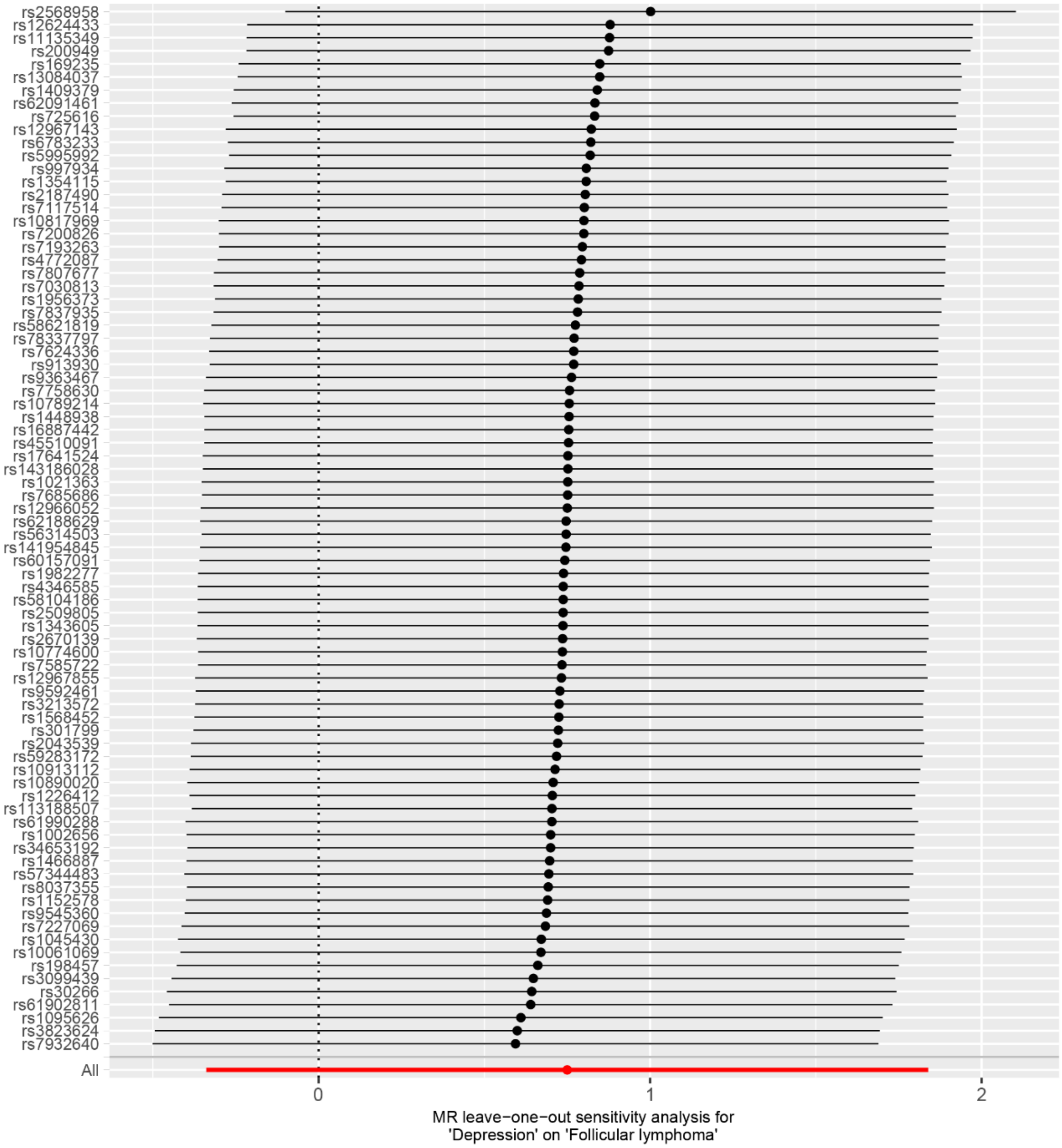

Figure S25: Leave-one-out inverse-variance weighted mendelian randomization analyses of depression on Follicular lymphoma

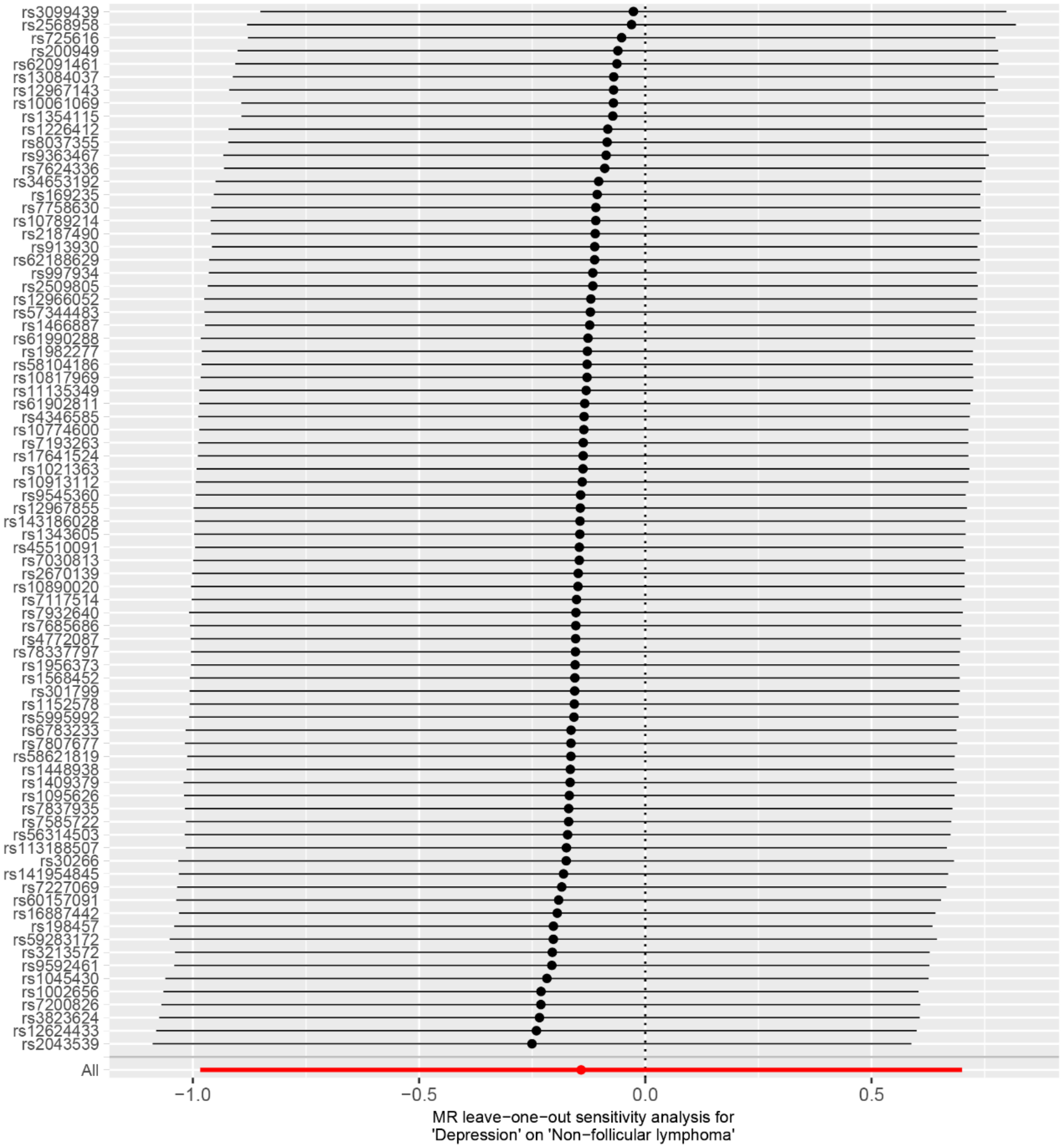

Figure S26: Leave-one-out inverse-variance weighted mendelian randomization analyses of depression on Non-follicular lymphoma

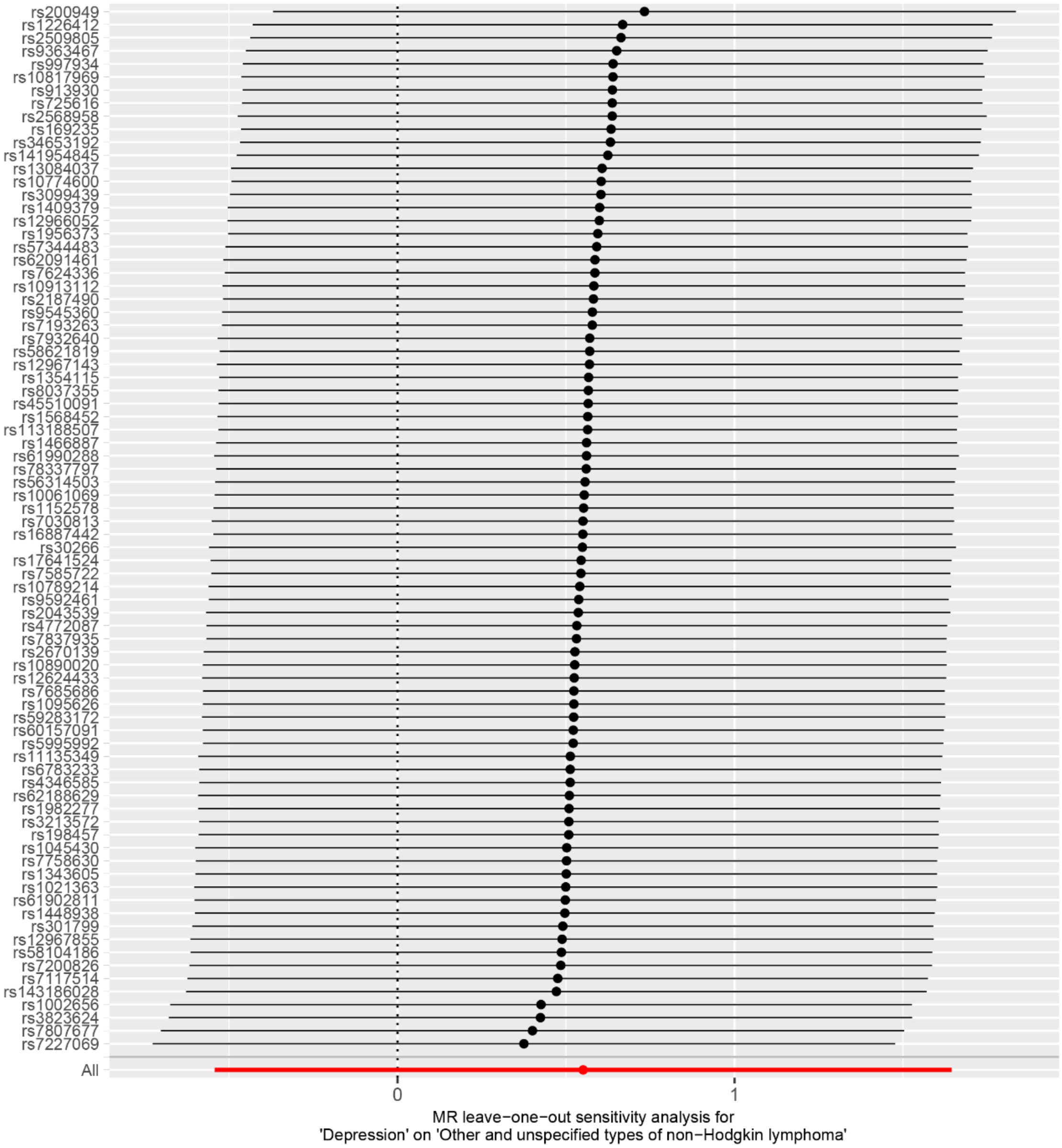

Figure S27: Leave-one-out inverse-variance weighted mendelian randomization analyses of depression on Other and unspecified types of non-Hodgkin lymphoma

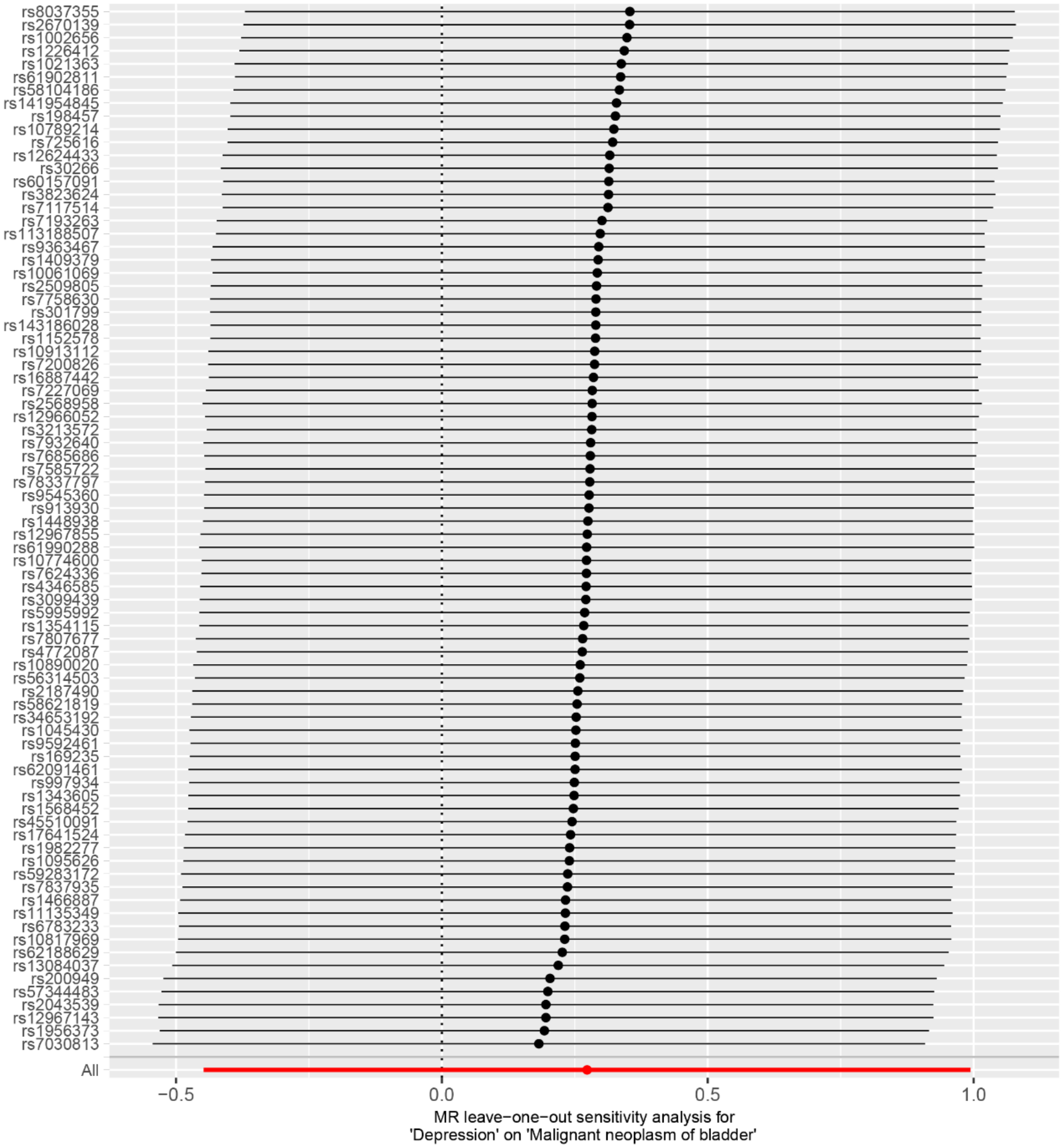

Figure S28: Leave-one-out inverse-variance weighted mendelian randomization analyses of depression on Malignant neoplasm of bladder

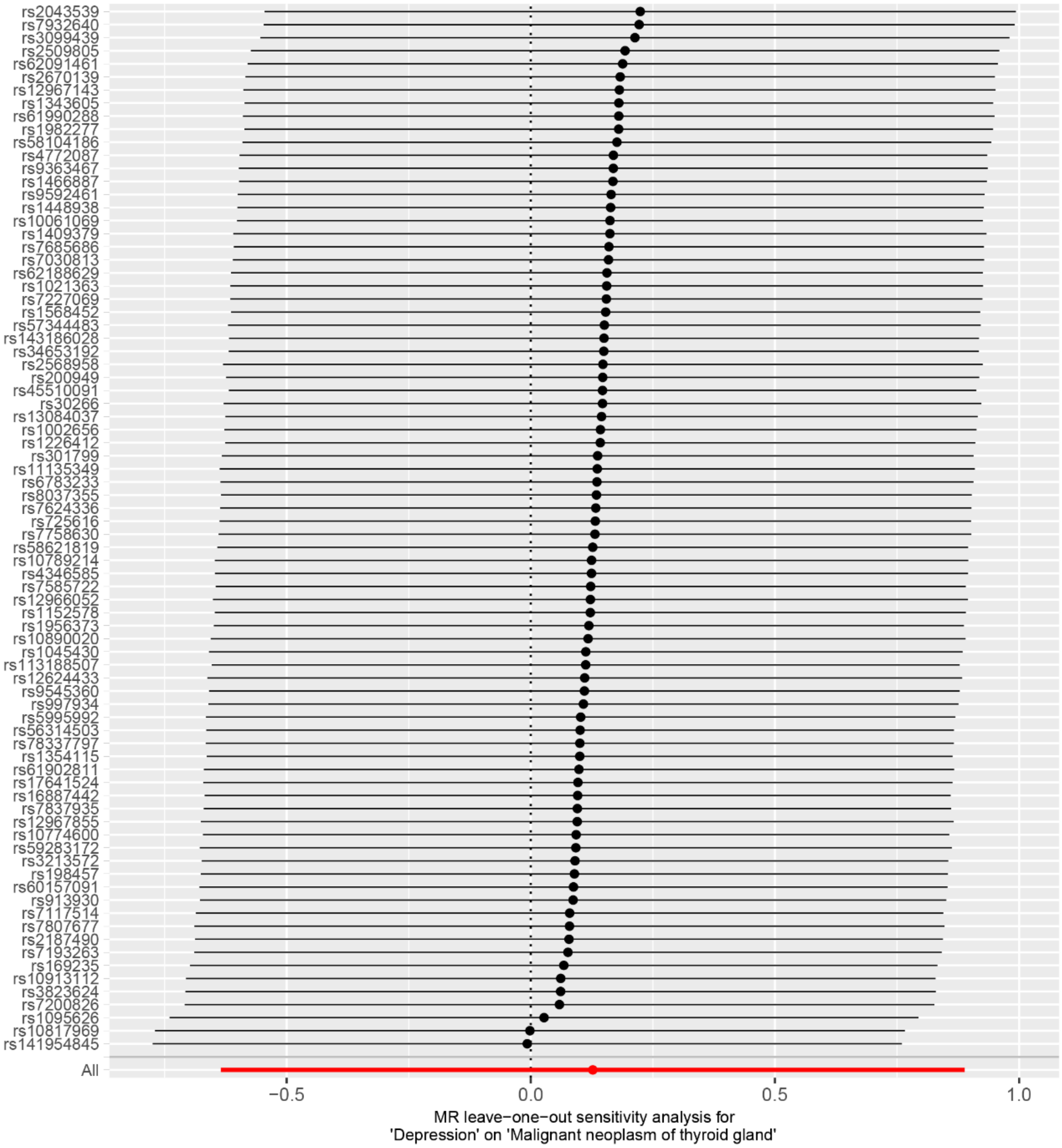

Figure S29: Leave-one-out inverse-variance weighted mendelian randomization analyses of depression on Malignant neoplasm of thyroid gland

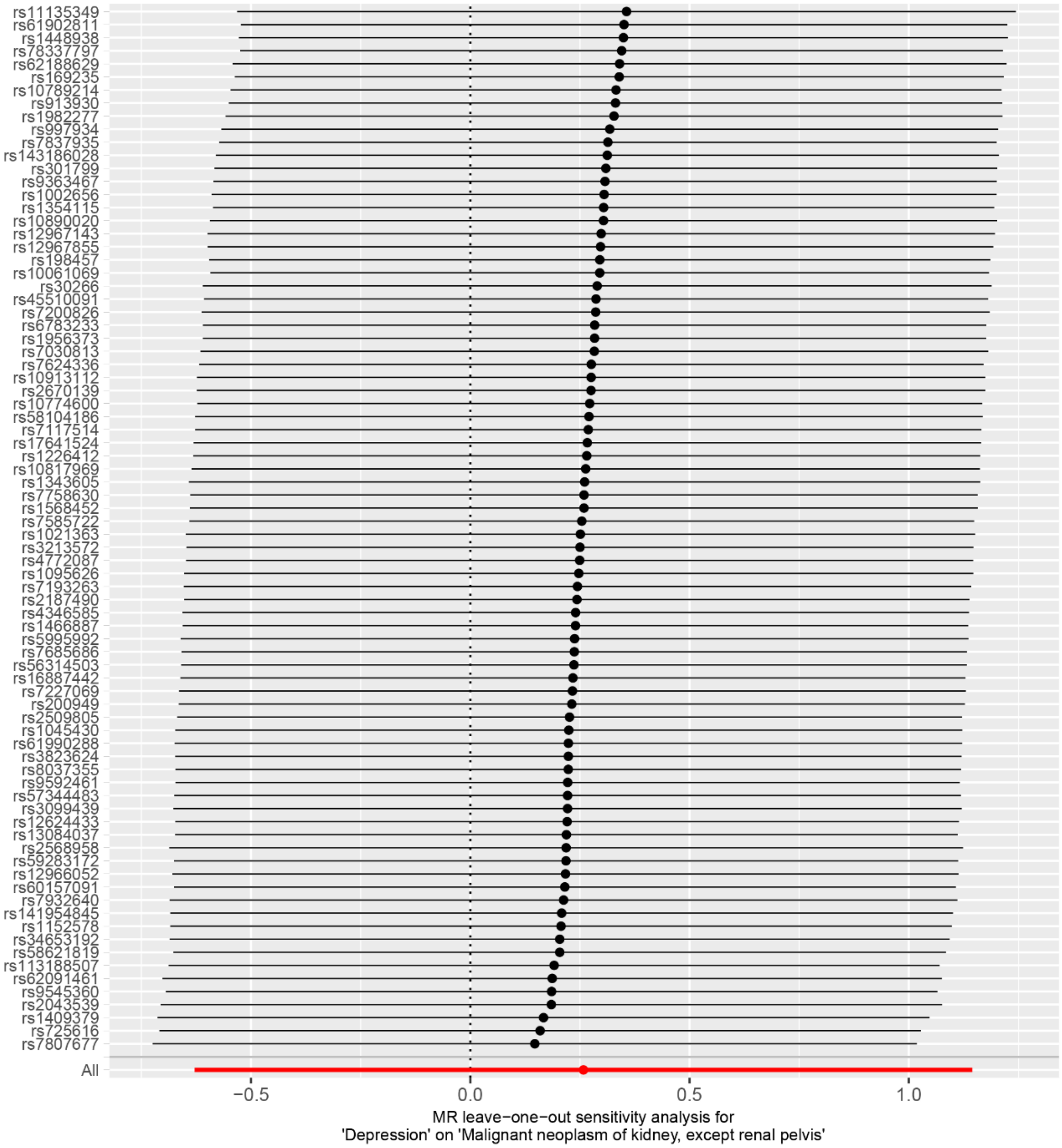

Figure S30: Leave-one-out inverse-variance weighted mendelian randomization analyses of depression on Malignant neoplasm of kidney, except renal pelvis

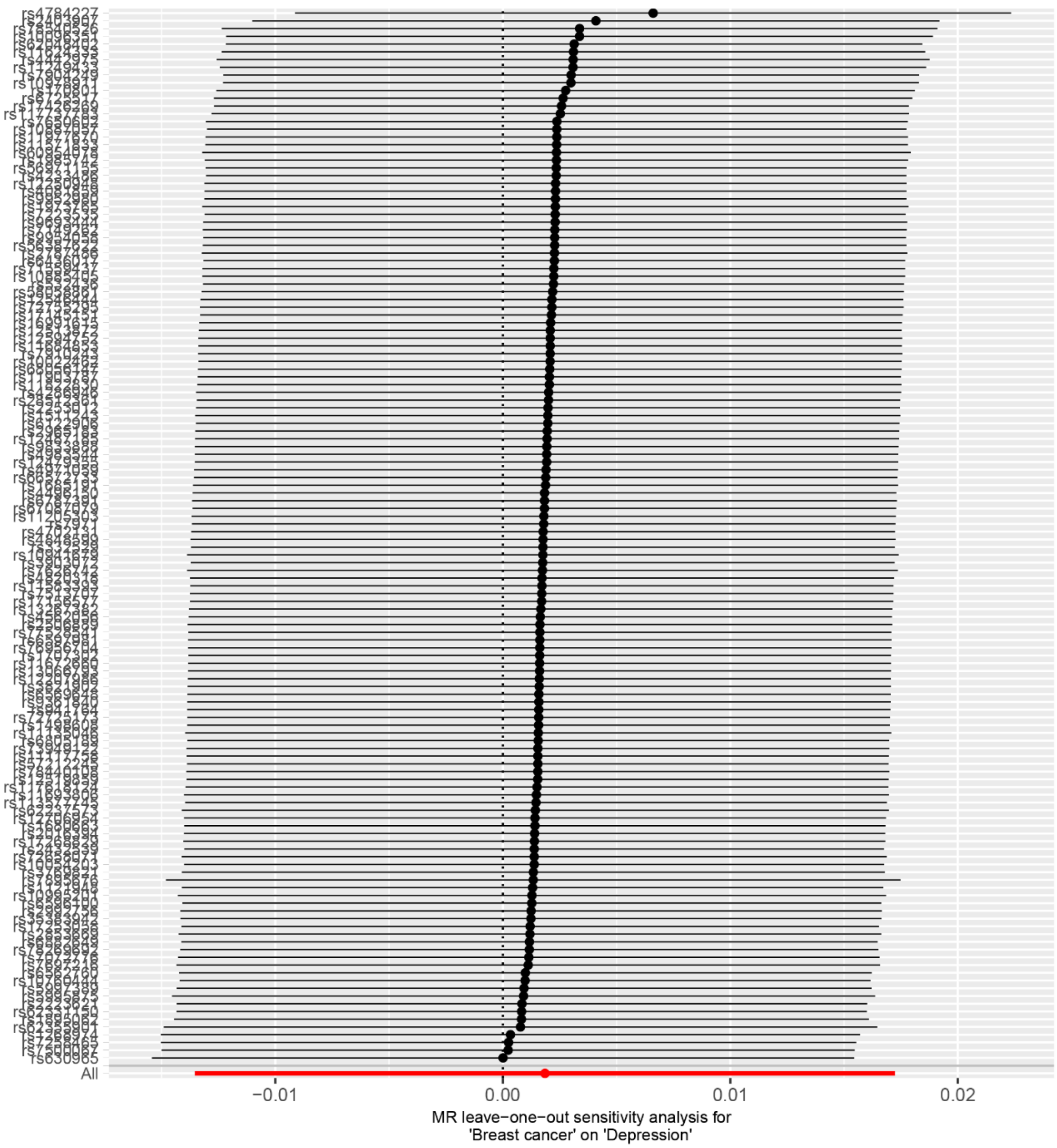

Figure S31: Leave-one-out inverse-variance weighted mendelian randomization analyses of Breast cancer on depression

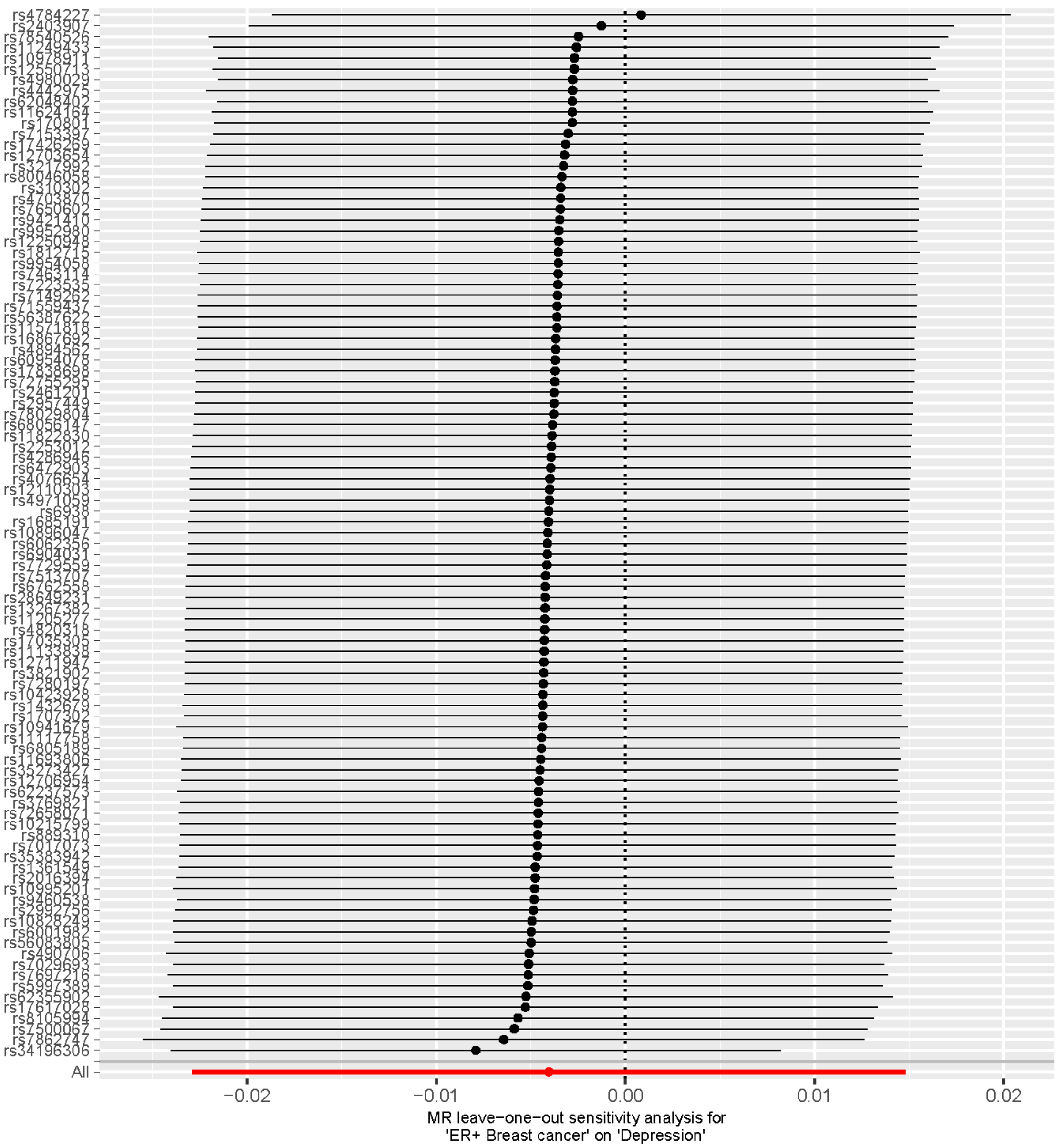

Figure S32: Leave-one-out inverse-variance weighted mendelian randomization analyses of ER+ Breast cancer on depression

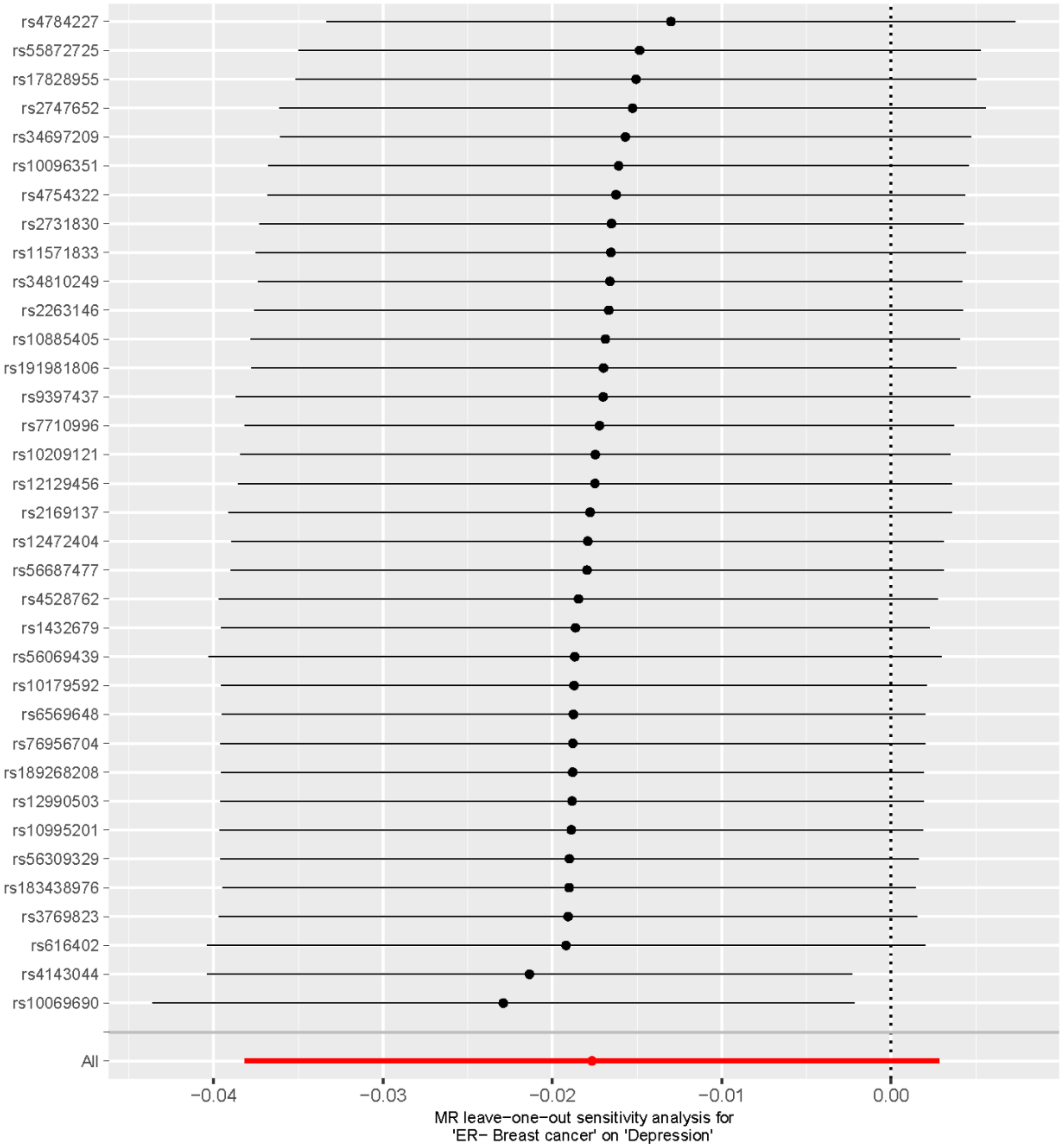

Figure S33: Leave-one-out inverse-variance weighted mendelian randomization analyses of ER- Breast cancer on depression

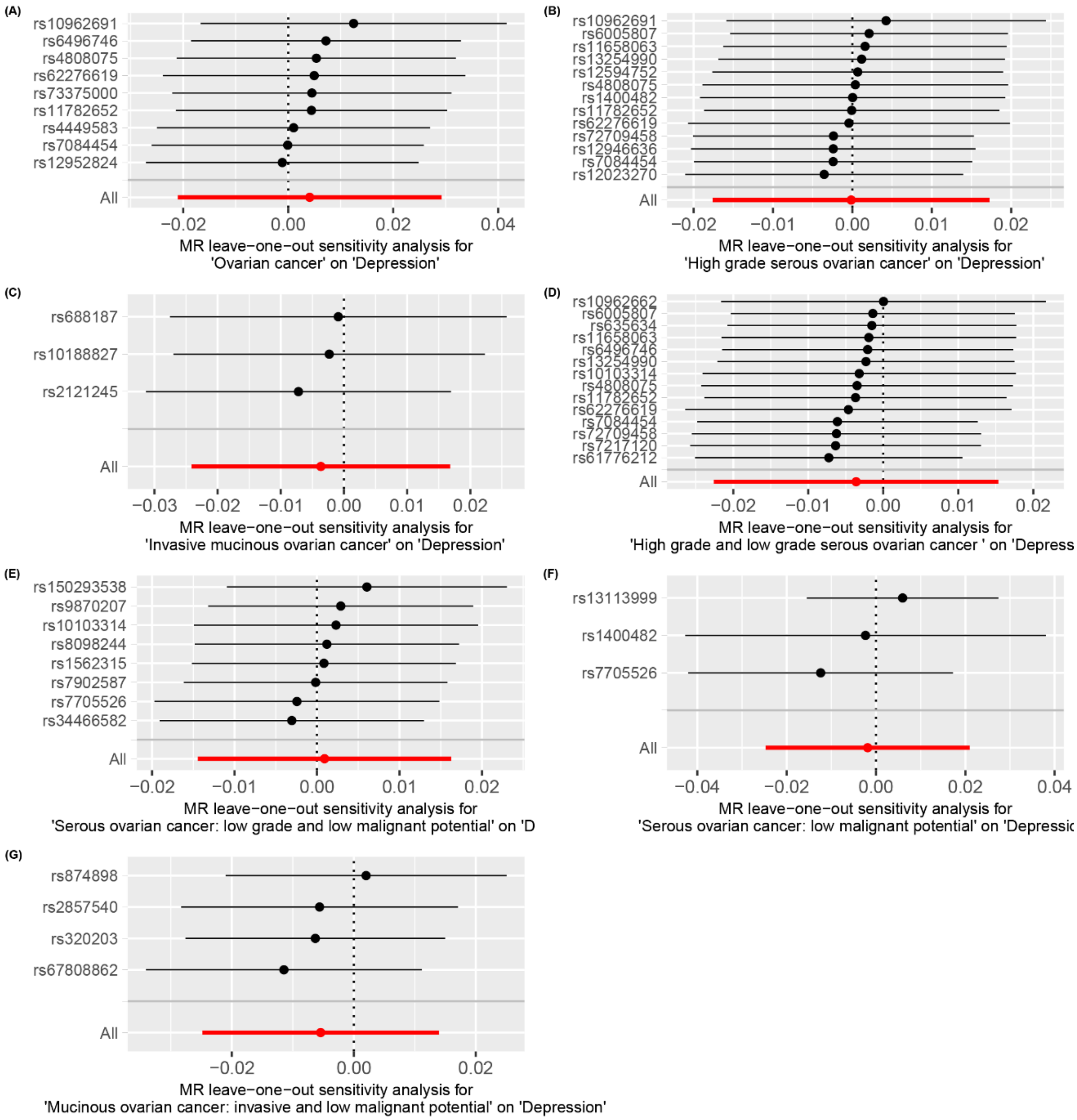

Figure S34: Leave-one-out inverse-variance weighted mendelian randomization analyses of different types of ovarian cancer on depression: (A) Ovarian cancer; (B) High grade serous ovarian cancer; (C) Invasive mucinous ovarian; (D) High grade and low grade serous ovarian cancer; (E) Serous ovarian cancer: low grade and low malignant potential; (F) Serous ovarian cancer low malignant potential; (G) Mucinous ovarian cancer: invasive and low malignant potential.

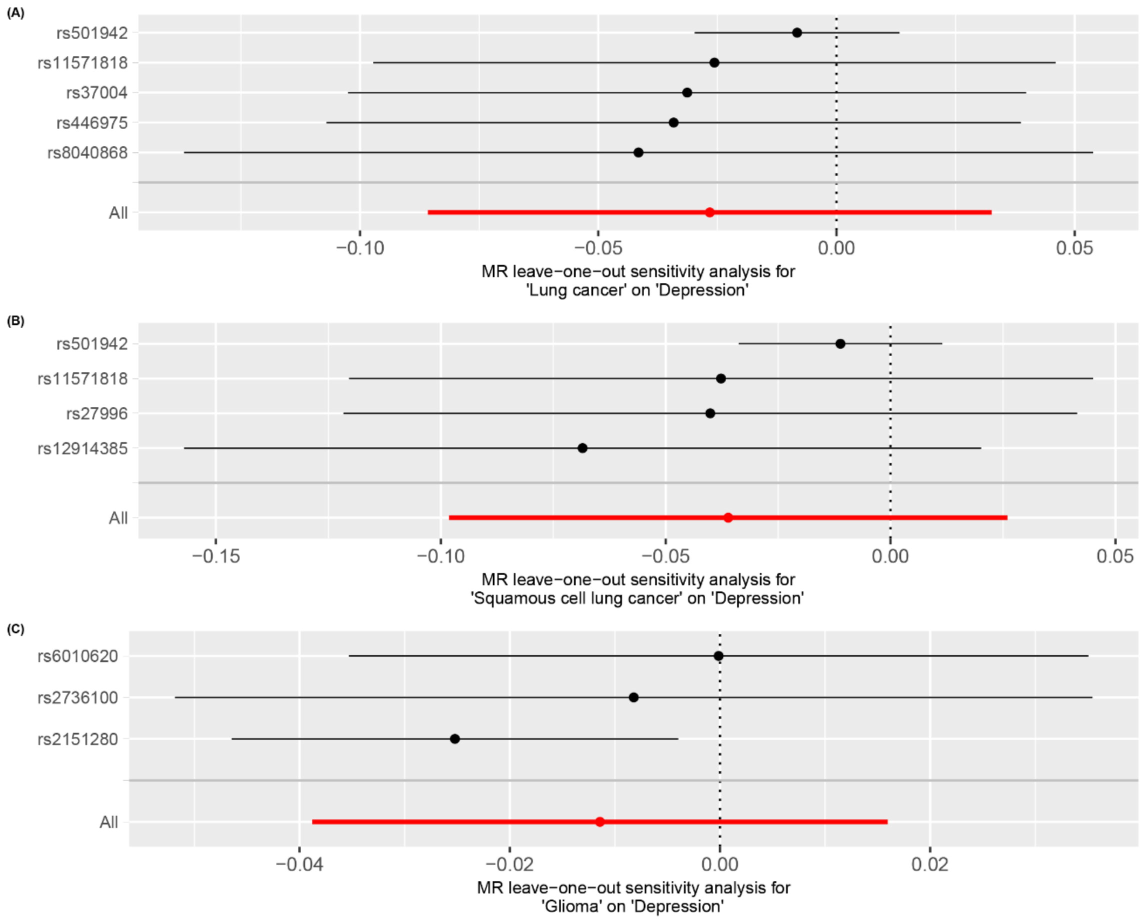

Figure S35: Leave-one-out inverse-variance weighted mendelian randomization analyses of different types of cancer on depression: (A) Lung cancer; (B) Squamous cell lung cancer; (C) Glioma.
